# CCR4-NOT Transcription Complex Subunit 7 (CNOT7) Protein and Leukocyte-Associated Immunoglobulin-like Receptor-1 in Breast Cancer Progression: Clinical Mechanistic Insights and In Silico Therapeutic Potential

**DOI:** 10.3390/ijms26157141

**Published:** 2025-07-24

**Authors:** Mona M. Elanany, Dina Mostafa, Ahmad A. Hady, Mona Y. Y. Abd Allah, Nermin S. Ahmed, Nehal H. Elghazawy, Wolfgang Sippl, Tadashi Yamamoto, Nadia M. Hamdy

**Affiliations:** 1 Department of Biochemistry, Faculty of Pharmacy, Ain Shams University, Abassia, Cairo 11566, Egypt; 2 Department of Clinical Oncology and Nuclear Medicine, Faculty of Medicine, Mansoura University, Dakahlia, Mansoura 35516, Egypt; 3 Department of Pathology, Faculty of Medicine, Mansoura University, New Cairo, Mansoura 35516, Egypt; monayounis@mans.edu.eg; 4 Department of Pharmaceutical Chemistry, Faculty of Pharmacy and Biotechnology, German University in Cairo, New Cairo, Cairo 11835, Egypt; 5 Department of Medicinal Chemistry, Institute of Pharmacy, Martin-Luther-University of Halle-Wittenberg, 06120 Halle, Germany; nehal.elghazawy@gmail.com (N.H.E.);; 6 Cell Signal Unit, Okinawa Institute of Science and Technology (OIST), Kunigami-gun, Okinawa 904-0495, Japan

**Keywords:** CNOT7, natural killer cell resistance, LAIR-1, metastatic breast cancer, in silico/bioinformatics analysis, molecular docking

## Abstract

Metastatic breast cancer (BC) spread underscores the need for novel prognostic biomarkers. This study investigated CCR4-NOT Transcription Complex Subunit 7 (CNOT7) and leukocyte-associated immunoglobulin-like receptor-1 (LAIR-1) in BC progression and natural killer (NK) cell resistance. In the current study, 90 female BC patients (46 non-metastatic, 44 metastatic) were analyzed. CNOT7 and LAIR-1 protein levels were measured in serum via ELISA and CNOT7 expression in tissue by immunohistochemistry (IHC). In silico tools explored related pathways. Computational analyses, including in silico bioinformatics and molecular docking, explored gene functions, interactions, and ligand binding to CNOT7 and LAIR-1. CNOT7 serum levels were significantly elevated in metastatic patients (mean 4.710) versus non-metastatic patients (mean 3.229, *p* < 0.0001). Conversely, LAIR-1 serum levels were significantly lower in metastatic (mean 56.779) versus non-metastatic patients (mean 67.544, *p* < 0.0001). High CNOT7 was found in 50% (45/90) of cases, correlating with higher tumor grade, hormone receptor negativity, and increased lymph node involvement. Elevated CNOT7 and lower LAIR-1 levels were associated with worse overall survival. Pathway analysis linked CNOT7 to the PI3K/AKT/mTOR pathway. Computational findings elucidated CNOT7′s cellular roles, gene/protein interaction networks for LAIR-1/CNOT7, and distinct ligand binding profiles. High CNOT7 levels are associated with advanced BC stages and poor clinical outcomes, which suggests its utility as a prognostic biomarker. The inverse relationship between CNOT7 and LAIR-1 provides mechanistic insights into BC progression and immune evasion, further supported by in silico investigations.

## 1. Introduction

### 1.1. Research Problem

Breast cancer (BC) is the primary cause of cancer deaths in women [1]. The main cause of this mortality is the metastatic spread to other organs [2]. Metastasis occurs when tumor cells acquire invasive features and the ability to escape from antitumor immunity, the innate and adaptive immune responses important for tumor control [3]. Major impairment of peripheral blood natural killer (NK) cell maturation and cytotoxic functions was reported to accompany BC progression [4]. Several gene expression profiling studies have shown that a better outcome is associated with a strong cytotoxic infiltrate containing NK cells [5,6]. These data suggest that BC progression is linked to the antitumor immune efficiency of NK cells, T-cells, and B-cells. However, how BC progression affects peripheral NK cells’ phenotype is not abundantly investigated.

The activity of NK cells is determined by activating and inhibitory receptors present on NK cells that are triggered during target cell (herein, tumor cell) recognition, inducing a positive or a negative cell signaling pathway, respectively. The integration of these opposite signals determines the NK cell activation threshold [7]. Recent studies have shown that several molecules, notably, inhibitory factors, often found in the tumor microenvironment (TME), can sharply impair NK cells’ phenotype and functions [8], resulting in a decreased expression of activating NK cell receptors and an increased expression of the inhibitory receptors (like LAIR-1, which was detected previously in hepatocellular carcinoma (HCC) clinical blood samples’ T-cytotoxic cells) [9]. These alterations may contribute to the progression of BC and could be associated with diminished cytotoxic function in immune cells like NK cells, T-cells, or B-cells [10]. Therefore, unraveling the molecular events in clinical BC tissue cases that may be responsible for peripheral mononuclear cells’ resistance represents a crucial step toward drug development or the repurposing of novel immunotherapies that should enhance NK cell functions.

CNOT7 is a key cytoplasmic mRNA deadenylase whose expression is altered in a variety of human cancers, which suggests its carcinogenic role in tumor development [11,12]. Recently, CNOT7 was found to be highly expressed in MCF7 cells (a human breast cancer cell line) relative to the normal BC cell line [13]. Furthermore, Faraji and coworkers found that higher expression of CNOT7 drives BC metastasis [14]. Beyond its role in cell growth, CNOT7 has also been shown to influence the tumor immune microenvironment and contribute to NK cell resistance. For instance, CNOT7 depletion can reverse NK cell resistance by suppressing immunosuppressive cytokines like transforming growth factor-β1 (TGF-β1) and promoting interferon-γ (IFN-γ) secretion by NK cells. This occurs, in part, through CNOT7′s regulation of nuclear factor-κB subunit p65 trafficking, impacting cytokine production and thereby influencing inhibitory signals that impair NK cell function [12]. Furthermore, knockdown of CNOT7 in MCF7 cells has been shown to overexpress signal transducer and activator of transcription 1 (STAT1), leading to the hyperactivation of STAT1-regulated genes and a subsequent inhibition or slowing of cell growth in both breast and liver cancer in vitro experiments [12,15]. These findings underscore the importance of investigating the clinical implications of CNOT7 in BC.

The present study aims to further elucidate the role of CNOT7 in immune cell resistance during BC metastasis, particularly in relation to LAIR-1. LAIR-1 is a key inhibitory receptor expressed on NK cells, and its engagement by collagen or other ligands in the TME can suppress NK cell cytotoxicity, contributing to immune evasion by tumor cells. CNOT7′s role in modulating the TME by altering cytokine profiles (e.g., reducing immunosuppressive TGF-β1) could indirectly influence the expression or signaling pathways of inhibitory receptors like LAIR-1 on NK cells. An immunosuppressive TME, potentially fostered by elevated CNOT7 activity, might lead to an upregulation or enhanced function of LAIR-1, thereby promoting NK cell anergy or exhaustion and contributing to tumor progression and immune evasion. Understanding this interplay is critical for identifying novel insights into BC metastasis mechanisms and potential targets for therapeutic intervention.

### 1.2. Aim and Objectives

Our study aimed to elucidate the role of CNOT7 in immune cell resistance during BC metastasis. We conducted a comprehensive investigation, beginning with the assessment of serum levels of CNOT7 in a cohort of Egyptian female BC patients and their correlation with clinicopathological parameters. Additionally, we examined the tissue expression of CNOT7 in BC samples categorized as metastatic or non-metastatic tumors, as well as in adjacent non-tumor tissue margins. Furthermore, we quantified the serum levels of the NK cell inhibitory receptor LAIR-1, known for its association with aggressive BC features and adverse clinical outcomes. We sought to establish a correlation between the levels of CNOT7 and LAIR-1 in the blood, clinically assessing their association with tumor-node-metastasis TNM staging. This correlation was further validated or negated through searches in bioinformatics databases and in silico analysis. Recognizing that our focus extends beyond exploring CNOT7 or LAIR-1 blood levels, our objective is to identify potential therapeutic avenues. This includes investigating CNOT7 molecular docking and exploring the feasibility of blocking its downstream pathways or interactions with effector genes, thereby offering promising treatment modalities. To achieve these objectives, we leveraged KEGG pathway databases to identify relevant pathways and interactions, supplementing this information with text-mined interactions and curated in silico bioinformatics databases. Through these comprehensive analyses, our goal is to unravel novel insights into BC metastasis mechanisms and identify potential targets for therapeutic intervention.

We anticipate that our findings will expand our knowledge of CNOT7’s role in NK resistance in BC clinically and identify CNOT7 as a potential future target hit for therapy.

## 2. Results

### 2.1. BC Patient Group Participants’ Demographic and Clinical Characteristics

This study included 90 patients with BC who were categorized into non-metastatic BC (*n* = 46) and metastatic BC (*n* = 44) groups that were compared to each other (Table 1).

### 2.2. CNOT7 and LAIR-1 Levels in Peripheral Blood Samples of Metastatic BC Patients Group Compared to the Non-Metastatic Group

**Figure 1:**
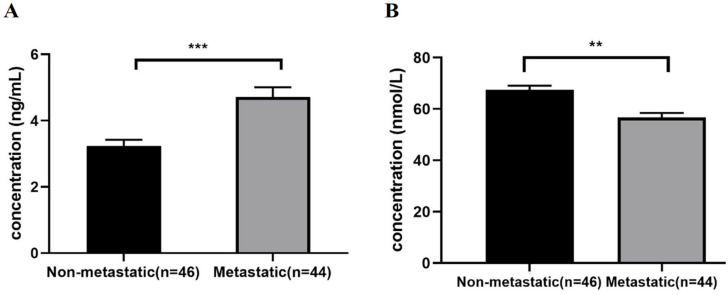
Serum levels of (**A**) CNOT7 and (**B**) LAIR-1 in non-metastatic (n = 46) compared to metastatic (n = 44) BC patients’ groups (measured in serum by ELISA). Data are presented as mean ± SEM, statistics computed using Student’s *t*-test for parametric data. ** Statistical significance is set at *p* < 0.01, *** Statistical significance is set at *p* < 0.001.

CNOT7 serum levels were significantly elevated (*p* < 0.0001) in the metastatic compared to the non-metastatic BC patients, with a mean ± SEM of 4.710 ± 0.296 and 3.229 ± 0.193, respectively, whereas LAIR-1 serum levels were significantly lower (*p* < 0.0001) in the metastatic compared to the non-metastatic BC patients, with a mean ± SEM of 56.779 ± 1.727 and 67.544 ± 1.582, respectively (Figure 1A,B).

### 2.3. CNOT7 Protein Expression in BC Patients’ Tissue Samples

**Figure 2:**
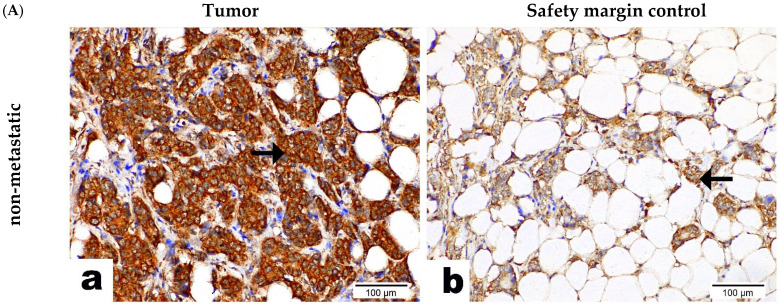

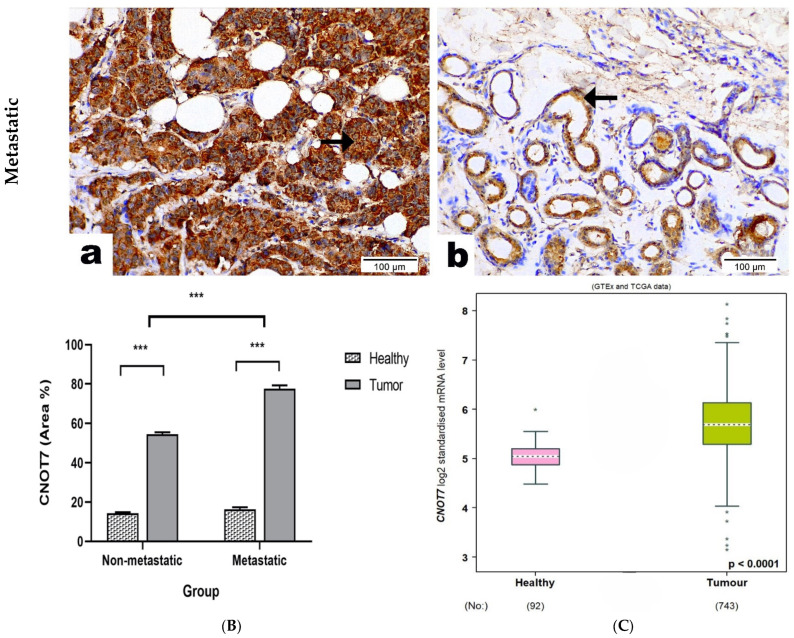
CNOT7 expression in the metastatic group compared to the non-metastatic BC tissues, detected via (**A**) IHC in breast tumor tissue (**a**) and its adjacent safety-margin healthy breast tissues (**b**). Cells with yellow-brown staining are immune-positive, and the arrows denote the intracellular localization of CNOT7. (Sections 100 um, Magnification ×400); (**B**) IHC quantitative analysis (mean ± SEM) showing an increased CNOT7 expression in BC tissue compared to the healthy tissue and increased CNOT7 expression in the metastatic (n = 4) compared to non-metastatic BC (n = 4). Comparison was performed using Student’s *t*-test. *** Statistical significance is set at *p* < 0.001; and (**C**) box plot interquartile range (IQR) of *CNOT7* mRNA expression levels (log2 standardized mRNA level) in healthy (n = 92) and tumor (n = 743) breast tissues * represent data more than 1.5 times the IQR; ** represent data more than 3 times the IQR. Data (**C**) were generated using bc-GenExMiner v5.0 using GTEx and TCGA data (http://bcgenex.ico.unicancer.fr/BC-GEM/GEM-Accueil.php?js=1) accessed on 8 May 2023.

Immunohistochemistry (IHC) staining was performed in paraffin-embedded metastatic and non-metastatic BC specimens, including healthy breast tissue (Figure 2A), as also shown in Supplementary Figure S2. CNOT7 was expressed predominantly in the cytoplasm and nucleus of all cancerous tissue, while its expression was membranous and cytoplasmic in the healthy breast tissue. CNOT7 expression was significantly higher in the cancerous tissue of the metastatic BC group compared to their corresponding non-metastatic BC group, while the expression of CNOT7 in healthy tissue was significantly lower than that in the cancerous tissue of both groups (Figure 2B). These results indicate that the high expression of CNOT7 may be related to the metastasis of BC. These results were confirmed by measuring CNOT7 mRNA expression levels using bc-GenExMiner v 4.5 (Figure 2C), showing a significant increase in CNOT7 mRNA levels in tumor tissue compared to healthy tissue (*p* < 0.0001) retrieved from databases.

### 2.4. LAIR-1 or CNOT7 Serum Levels’ Association with the Clinicopathological Data of the BC Cohort (n = 90)

According to the cut-off value obtained from the ROC curve, high serum levels of LAIR-1 (more than 61.01) were seen in 54.4% of cases (49/90). High LAIR-1 serum levels were associated with hormone receptor positivity, early tumor stages, and non-diabetic status, as shown in Table 2, and Figure S3 presents significant associations between LAIR-1 serum levels (low and high) and clinicopathological variables in BC patients (*n* = 90).

Again, according to the cut-off value obtained from the ROC curve, high serum levels of CNOT7 (>3.62) were seen in 50% of cases (45/90). High CNOT7 serum levels were associated with BMI, post-menopausal status, receiving neoadjuvant chemotherapy before surgery, late tumor stages (III/IV), greater lymph node (LN) involvement, basal and human epidermal growth factor receptor 2 (HER-2)-enriched subtypes, and estrogen receptor (ER) and progesterone receptor (PR) hormone receptor negativity (Table 2). Therefore, high serum CNOT7 levels are associated with advanced BC stages and hormone receptor status, as presented in Figure S4, which presents significant associations between CNOT7 serum levels (low and high) and clinicopathological variables in BC patients (*n* = 90).

CNOT7 expression across different BC subtypes has also been retrieved from publicly available datasets (Figure S6), where a significantly higher expression of CNOT7 was associated with grade 3 BC, basal molecular subtype, and ER and PR receptor negativity, supporting the data obtained from our cohort.

### 2.5. Correlations Results

The correlations between CNOT7 serum levels and the clinicopathological data of the BC cohort are summarized in Table 3 and Supplementary Figure S6. High CNOT7 serum levels were correlated with post-menopausal status, negative ER status, higher LN involvement, and late TNM stages.

### 2.6. CNOT7 Correlation with LAIR-1

**Figure 3:**
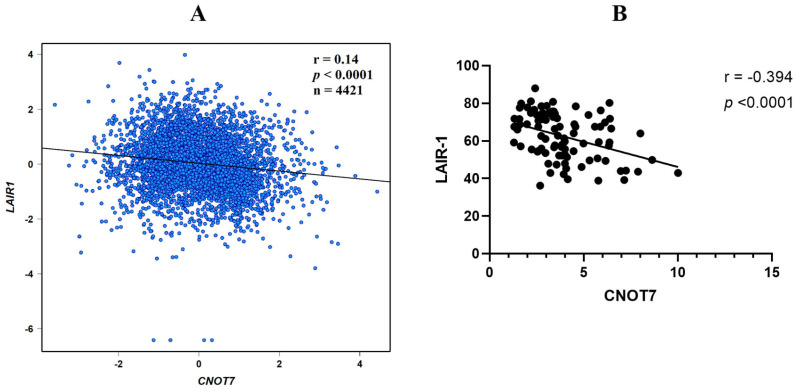
Pearson pairwise correlation plot for CNOT7 vs. LAIR-1 (**A**) data generated using the bc-GenExMiner v 4.5 *(n* = 4421) (http://bcgenex.ico.unicancer.fr/BC-GEM/GEM-Accueil.php?js=1), accessed on 8 May 2023; and (**B**) in the current patients’ cohort (*n* = 86).

To examine whether CNOT7 contributes to metastasis via affecting the expression of the NK cell inhibitory receptor LAIR-1 or not, Pearson correlation analysis was performed. This association was confirmed, first, from the bc-GenExMiner v5.0 bioinformatics database results (Figure 3A) showing a negative correlation between both markers, with r = −0.14, *p* < 0.0001, *n* = 4421. In our current cohort of BC patients, a significant negative correlation was observed between CNOT7 and LAIR-1 serum levels, with a correlation coefficient of r = −0.394 and *p* < 0.0001 (Figure 3B).

### 2.7. Prognostic Value of CNOT7 and LAIR-1 Serum Levels

**Figure 4:**
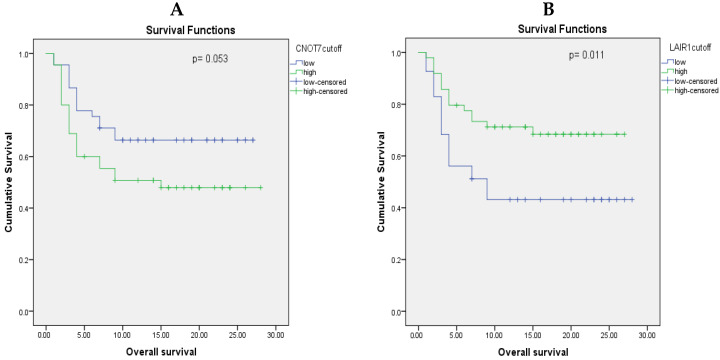
Kaplan–Meier survival curves for BC samples. (**A**) Low (green line) vs. high (blue line) CNOT7 serum levels and (**B**) low (green line) vs. high (blue line) LAIR-1 serum levels.

To evaluate how CNOT7 and LAIR-1 levels impact the survival of BC patients, we conducted a Kaplan–Meier survival analysis. Patients were divided into “high” and “low” groups for both CNOT7 and LAIR-1 based on the median serum level of each marker. Our analysis revealed that BC patients with high CNOT7 serum levels had a significantly shorter estimated survival time compared to those with lower CNOT7 levels (*p* = 0.053, Figure 4A). Conversely, lower LAIR-1 serum levels were significantly associated with shorter survival times when compared to higher LAIR-1 levels (*p* = 0.011, Figure 4B). These findings suggest that both CNOT7 and LAIR-1 serum levels can serve as indicators of BC patient prognosis.

### 2.8. Molecular Docking Results

Following the preparation of LAIR-1 and CNOT7 receptors in the MOE, six ligands, namely, vitamin E, colchicine, prodigiosin, riboflavin, telmisartan, and pioglitazone, were docked. (These ligands are chosen for being studied previously or currently by the research PI for their immuno-modulatory effect being repurposed for cancer treatment.)

**Figure 5:**
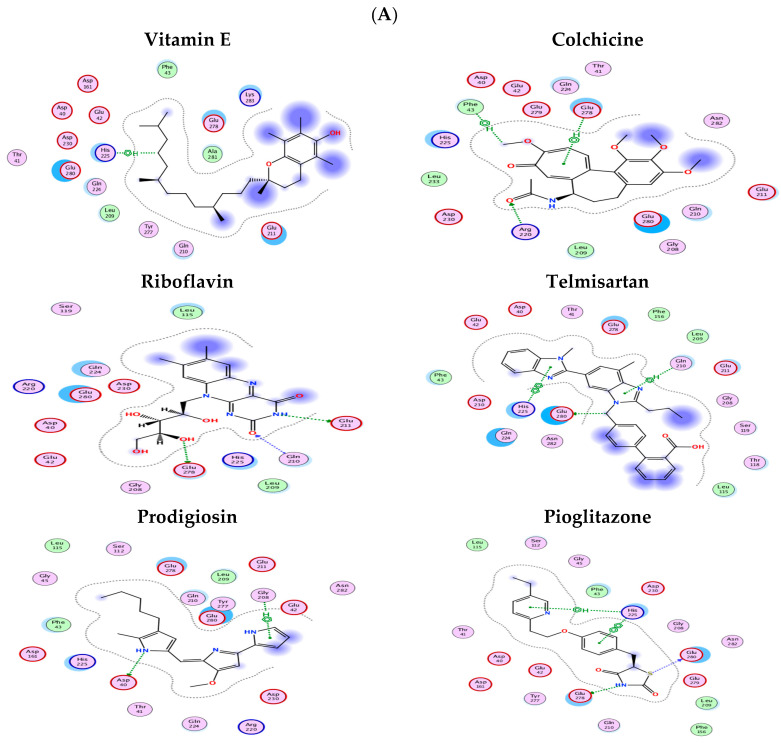

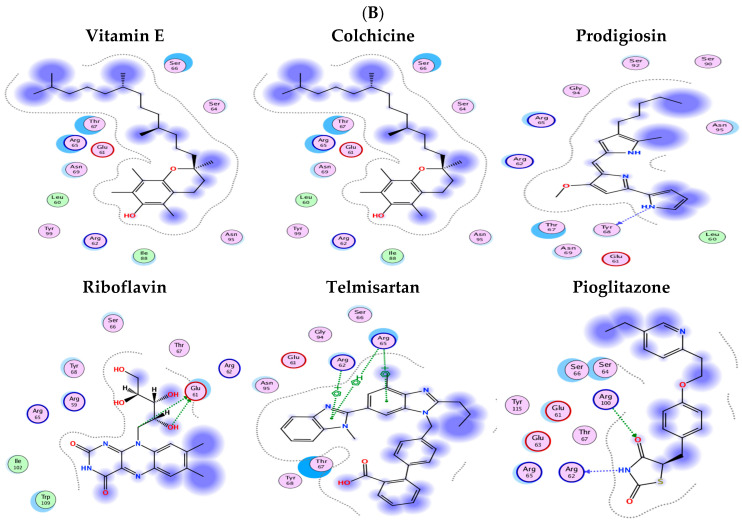
(**A**) **All ligands binding to CNOT7 binding pocket**, where prodigiosin interacted with Asp40 and Gly208; vitamin E interacted with His225; telmisartan had a π-π interaction with His22 and a CH/π interaction with Gln210; riboflavin had multiple H-bonds with Gln210, Glu211, and Glu278; colchicine interacted with Glu278 via a CH/π interaction and via a H-bond with Arg220 and had a CH/π interaction with Phe43; and pioglitazone formed two π interactions with His225, one H-bond with Glu278, and another H-bond with Glu280. (**B**) **All ligands binding to LAIR-1 binding pocket**, where vitamin E showed no significant interaction; prodigiosin showed a single H-bond with Tyr68, a part of the determined pocket; pioglitazone made a H-bond with Arg62 and Arg100; riboflavin showed interactions with Glu61 that maintained two hydrogen bonds; and telmisartan and colchicine made multiple cationic–π interactions with Arg62 and Arg65.

In the CNOT7 docking studies, nearly all ligands interacted with at least one of the amino acids, except for prodigiosin, which only interacted with Asp40 and Gly208. Vitamin E formed a single CH/π interaction with His225, while telmisartan exhibited both a π-π interaction with His22 and a CH/π interaction with Gln210. Riboflavin, by contrast, formed multiple hydrogen bonds with Gln210, Glu211, and Glu278. Colchicine showed interactions with Glu278 through a CH/π interaction, a hydrogen bond with Arg220, and another CH/π interaction with Phe43. Lastly, pioglitazone formed two π interactions with His225, as well as hydrogen bonds with Glu278 and Glu280 (Figure 5A).

The possible ligand binding sites in the deposited LAIR-1 crystal structure (PDB ID: 3RP1) were identified using MOE SiteFinder. The site was selected based on the descending order of the PLB score. Moreover, reported mutational analysis has identified some residues as being involved in the interaction with LAIR-1, such as Arg59, Glu61, Glu72, Ile102, Pro107, and Glu111 [16]. Accordingly, the binding pocket was selected to be the highest-ranking pocket suggested by MOE SiteFinder, along with the rest of the reported amino acids.

All ligands were able to bind to the LAIR-1 binding pocket (Figure 5B)**,** except for vitamin E, which showed no significant interaction. Prodigiosin formed a single hydrogen bond with Tyr68, a residue within the pocket identified by MOE SiteFinder. Pioglitazone, interestingly, established hydrogen bonds with Arg62 and Arg100, both key residues. Riboflavin also interacted with crucial amino acids reported in the literature, forming two hydrogen bonds with Glu61. Lastly, telmisartan and colchicine demonstrated multiple cation–π interactions with Arg62 and Arg65, a crucial residue in LAIR-1 interactions [17].

## 3. Discussion

CNOT7 is a member of the Asp-Glu-Asp-Asp residues (DEDD) superfamily of deadenylases and one of the CCR4-NOT deadenylase complex subunits [18]. Its gene is widely expressed in tissues of adult animals [19]. High expression of CNOT7 has been reported to be a significant factor in the development of various cancers [12,20,21,22]. Several reported observations led us to investigate the role of CNOT7 in BC progression and metastasis. In vivo, CNOT7 knockdown reduced pulmonary metastasis but had no effect on initial mammary tumor development, which indicates that its role in tumor progression is tied to the metastatic process [14]. CNOT7 was also revealed to have a role in the proliferation and migration of MCF7 cells [13,23]. This is consistent with our findings, which indicate an increase in serum levels and tissue expression levels by IHC of CNOT7 in metastatic BC patients compared to non-metastatic patients. We also showed that CNOT7 is associated with aggressive features of BC and adverse clinical outcome, as higher CNOT7 levels were significantly correlated with late stages of BC; more aggressive subtypes, i.e., TNBC and HER-2+; and greater LN involvement. Moreover, we observed an almost significant positive association between diabetes mellitus and high expression of CNOT7; this result is consistent with a previous study stating that CNOT7 knockdown enhanced insulin sensitivity and glucose clearance [24].

Given that NK cells play a crucial role in the innate immune response against cancer, exerting potent anticancer and antimetastatic effects, our understanding of how BC progression influences the phenotype of peripheral NK cells is not abundantly investigated [25]. One possible mechanism of how BC progression affects peripheral NK cells’ phenotype is that BC cells might upregulate NK cell inhibiting receptors or downregulate NK cell activating receptors. Although our study and others suggest the involvement of CNOT7 in BC metastasis, the specific biological functions and molecular targets of CNOT7 in BC remain inadequately explored. Previous research in HCC has linked CNOT7 overexpression to resistance in NK cells [12]. This was evidenced by elevated CNOT7 expression levels correlating with the increased secretion of TGF-β1 by HCC cancer tissue, subsequently leading to reduced IFN-γ secretion by NK cells. TGF-β1 is a powerful immune suppressor secreted from cancer cells of various cancer types, including HCC [26]. IFNs are a group of pleiotropic cytokines in the tumor-suppressive network [27]. STAT1 is the first STAT protein identified in the IFN signal transduction pathways, which have an essential role in suppressing tumor cell proliferation, differentiation, apoptosis, and angiogenesis. CNOT7 overexpression in HCC tissues has also been found to be associated with decreased STAT1 expression, which normally forms a dimer with p65, a distinct protein of the NF-κB family of dimeric transcription factors, inhibiting it from being translocated to the nucleus to promote TGF-β1 transcription [28]. Along similar lines, our hypothesis posits that CNOT7 overexpression in BC may induce NK cell resistance, thereby promoting BC progression and metastasis. To identify potential molecular targets for CNOT7 on NK cells, we investigated the serum levels of LAIR-1, a pertinent inhibitory receptor expressed in a vast majority of NK cells (>90%) [29], and its high expression is associated with poor clinical outcomes in BC [30]. Our results are in line with a previous study reporting the association of LAIR-1 expression with luminal breast cancer. When LAIR-1 binds to collagen produced by osteoblasts, it triggers inhibitory signaling in natural killer (NK) cells, resulting in immune suppression. This immune suppression contributes to the survival of bone tumor cells in luminal breast cancer cases with bone metastasis [31].

Our data revealed a negative correlation between CNOT7 and LAIR-1, which does not agree with our initial hypothesis that the overexpression of CNOT7 is associated with an increased LAIR-1 expression negatively regulating NK cell function. The decreased serum levels of LAIR-1 in metastatic BC in our study patients’ cohort can be explained by the fact that LAIR-1 is not only expressed on lymphocytes, serving an inhibitory receptor function contributing to the tumor immunosuppressive microenvironment (TIME), but also LAIR-1 is overexpressed on cancer cells, playing a negative role in cancer progression. In line with our findings, LAIR-1 activation has been previously depicted to reduce the proliferation of myeloid leukemia cell lines [32] and acute myeloid leukemia (AML) blasts [33]. Additionally, LAIR-1 expression varies in chronic lymphocytic leukemia (CLL); it is absent in high-risk CLL, differently expressed in intermediate- and low-risk CLL, and significantly lower in CLL patients compared to healthy donors, correlating with disease stage and progression [34]. LAIR-1 expression was described in many solid cancers [35,36]. Wu et al. demonstrated that LAIR-1 expression was observed in HCC tissue and tumor-adjacent ones but not in normal liver tissue [36]. Similarly, LAIR-1 expression was detected in the tumor cells of ovarian cancer tissues and epithelial ovarian cancer (EOC) cell lines but not in normal ovarian tissues. In high-grade EOC, LAIR-1 expression was weak, and its downregulation in EOC cells resulted in increased cell proliferation, colony formation, and cell invasion. These findings suggest the potential involvement of LAIR-1 in solid tumor progression or metastasis. Conversely, LAIR-1 expression suppressed the proliferative ability of human cervical cancer cells and induced apoptosis [37].

LAIR-1 was identified as having an inhibitory effect on the tumor cells’ invasion ability, negatively affecting BC lesions’ elasticity value, which is an indication of cancer cells’ active growth and proliferation [38]. These findings suggest that LAIR-1 can be involved in solid tumor progression or metastasis, having a negative, rather than a positive, regulatory role in tumor biology. Our serum measurement of LAIR-1 might be reflective of this role, and, therefore, LAIR-1 expression in BC tissue is recommended to be examined clinically (a limitation to be addressed in future studies).

Another interesting finding in our study was the association between the serum level of each biomarker with overall survival (OS), where higher levels of CNOT7 were associated with worse OS while lower LAIR-1 levels were accompanied by lower OS. This was confirmed with Kaplan–Meier survival curves; thus, we can conclude the possible utility of our biomarkers as predictors of patients’ survival and outcomes.

We performed an in silico search and bioinformatics analysis to obtain further insights on CNOT7 and LAIR-1 proteins’ interaction and the possible biological or molecular mechanism(s) or pathways in which they may be involved. Significant GO term annotation by GSEA showed that CNOT7 may possibly play an extensive role in the progression of BC through its deadenylation activity. PPI pathways from curated databases and text mining revealed the activation of protein tyrosine phosphatases (PTPs), including PTPN6 and PTPN11, by LAIR-1 [39]. These phosphatases collaborate with protein tyrosine kinases (PTKs) to regulate reversible tyrosine phosphorylation processes. Specifically, PTPN6 and PTPN11 are involved in the phosphoinositide 3 kinase/AKT/mammalian target of rapamycin (PI3K/AKT/mTOR) signaling pathway [40], which has been implicated in cancer progression and metastasis [41]. Activation of this pathway promotes BC cell growth and tumor propagation [42], which makes it a potential therapeutic target for BC treatment [43].

Furthermore, the current study utilized an extensive computational method to forecast the interactions between CNOT7 and LAIR-1 with six candidate drugs (prodigiosin, vitamin E, telmisartan, riboflavin, colchicine, and pioglitazone) that are known to affect BC, using molecular docking techniques [44,45,46,47,48,49,50], respectively. The docking studies have provided insightful information on their potential interactions and binding affinities. The results highlight significant variations in the binding profiles of these ligands (like colchicine on tubulin), suggesting their cancer-modulatory effect, which could be linked to their chemical structures and the nature of the binding pockets within the receptors.

For the CNOT7 receptor, most ligands demonstrated at least one interaction with the amino acids previously reported in the literature, which indicates that these ligands could potentially influence the receptor’s function. Notably, riboflavin showed multiple hydrogen bonds with key residues such as Gln210, Glu211, and Glu278, which suggests a strong binding affinity. This could imply a potentially significant effect on CNOT7′s biological activity. Colchicine, with its H-π interaction with Phe43 and Glu278, and pioglitazone, which formed multiple interactions, also exhibited strong binding. These interactions suggest that these ligands may have notable effects on CNOT7-related pathways, potentially offering therapeutic benefits.

By contrast, prodigiosin showed limited interaction, binding only with Asp40 and Gly208. This limited interaction may indicate a lower binding affinity or a different mode of action that does not primarily involve these residues. Similarly, vitamin E exhibited only a single interaction with His225, which suggests a weak binding affinity to CNOT7. These findings could be significant in understanding the varying degrees of influence these ligands may have on CNOT7 activity.

For the LAIR-1 receptor, the docking results were equally revealing. All ligands, except for vitamin E, demonstrated binding within the identified binding pocket. Riboflavin stood out for its interaction with key residues, maintaining hydrogen bonds with Glu61, which is crucial for LAIR-1’s function. This suggests that riboflavin could have a significant modulatory effect on LAIR-1 activity. Pioglitazone also showed a strong binding profile by forming hydrogen bonds with Arg62 and Arg100, key residues for LAIR-1 interaction, which suggests that it could play an influential role in LAIR-1-related pathways.

Telmisartan and colchicine, with their multiple cation–π interactions involving Arg62 and Arg65, further emphasize the potential of these ligands to modulate LAIR-1 activity. The interaction of these ligands with crucial amino acids indicates a strong binding affinity, which could translate into significant biological effects.

Interestingly, vitamin E did not show any significant interaction with LAIR-1, which suggests that it may not be effective in targeting this receptor or it may require a different binding site that was not explored in this study.

## 4. Subjects

### 4.1. Sample Size and the Power Study

The sample size estimation was performed using the G power* sample size online calculator accessed on 10 November 2021 (https://riskcalc.org/samplesize/#), depending on a two-sided significance level of 0.05 and power (1-beta) of 0.95.

Based on the previous study by Xue et al. [51], the standard deviations (SDs) were 3.2 and 3 with expected means of 4.6 and 3.9 for the diseased and control groups, respectively, and large effect size (1.2). From which, the current study groups’ calculated sample sizes were 44 for the metastatic and 46 for the non-metastatic BC patients. These sample sizes reject the null hypothesis that the population means of the studied groups are equal, with a probability (power) of 0.9.

### 4.2. Study Design

Mechanistic, retrospective, case-controlled, mono-center study.

#### Clinical Trial Registration

The trial is registered with https://www.clinicaltrials.gov/, identifier: NCT06320392.

### 4.3. BC Patients’ Groups

Patients included 90 female BC patients who volunteered for this study, diagnosed with either primary (non-metastatic) invasive BC (46 patients) or metastatic BC (44 patients). Patients were enrolled randomly from the Clinical Oncology Department, Faculty of Medicine, Mansoura University Hospitals, Mansoura University, Mansoura, Egypt, after signing the IC.

BC diagnosis was carried out with mammogram and magnetic resonance imaging (MRI). Full history was collected and recorded for all study participants (*n* = 90).

Blood samples and the tissue paraffin blocks were collected for those who met the inclusion criteria and signed the IC.

#### Patients’ Inclusion Criteria

Included patients encompassed adults over 18 years old with breast carcinoma confirmed pathologically.

#### The Exclusion Criteria

Excluded patients comprised patients with blood disorders, any cancer other than BC, liver cirrhosis, or uterine or urinary bladder diseases. Finally, patients with incomplete data or incomplete histopathology diagnosis reports were excluded.

### 4.4. Patients’ Clinical and Pathological Features

For all BC participants, full family disease/cancer history was recorded, as well as patients’ diabetic status (yes vs. no), number of pregnancies (either less or more than 2), menopausal status (pre vs. post), and treatment(s) received (neoadjuvant chemotherapy, adjuvant chemotherapy, endocrine therapy, or mastectomy). Patients’ individual current cancer status and the tumor clinical assessment, performed at the Faculty of Medicine, Mansoura University Hospital, Mansoura University, using the TNM categorization according to the 8th edition of the American Joint Committee on Cancer (AJCC) staging manual and the Bloom–Richardson Scale for histological grading [52], were collected from patients’ data files after a biopsy was taken at the time of BC surgical exploration/examination.

IHC results for proliferation index Ki-67 (being either low or high), estrogen receptor ER, and PR status and HER-2/neu status (each either positive or negative), BMI in kg/m^2^ (normal, overweight, and obese: 18.5–24.9, 25–29.9, and ≥30 kg/m^2^, respectively) [53], LN status (none N0, N1–3, N4–9, ≥10) [54], and age in years were all collected from patients’ files and tabulated for statistical analysis.

Molecular BC subtype [55] if luminal-like (ER+ and/or PR+), HER-2 overexpression (ER-, PR-, HER-2+), and triple-negative BC (TNBC) (ER-, PR-, HER-2-) and histological BC subtypes [56], whether invasive ductal carcinoma (IDC) or not, were all recorded for correlation analysis.

## 5. Materials and Methods

### 5.1. Blood Samples

Four milliliters of peripheral venous blood was withdrawn once from each patient, under strict sterile conditions, following standard safety procedures, into polymer gel vacutainers with a clot activator (Greiner Bio-One GmbH, Kremsmünste, Australia) and left for 15 min at room temperature to clot, followed by a 10 min centrifugation at 10,000× *g* at 4 °C. Sera obtained after centrifugation were aliquoted into three clean Eppendorf tubes and stored at −80 °C.

### 5.2. Paraffin Sections Tissue Samples

The tumor was fixed in NBF (10% neutral buffered formalin fixative) for 24 h at room temperature before being embedded in paraffin using a TissueTek VIP automated processor (Sakura Finetek, Torrance, CA, USA) in accordance with the standard protocol used in the laboratory for breast tumor diagnosis and staging at Mansoura University Hospitals. This procedure was carried out overnight at room temperature and consisted of dehydration in 100% ethanol (four baths totaling 3 h), butanol for 30 min, toluene (three baths totaling 2 h), and paraffin immersion (four baths at 58 °C totaling 3 h).

### 5.3. Biochemical Analysis

#### 5.3.1. Human CNOT7 or LAIR-1 ELISA (Mybiosource, San Diego, CA, USA)

Following the manufacturer’s instructions, the standards and serum samples of both non-metastatic and metastatic BC groups were incubated with CNOT7 or LAIR-1 HRP (Horseradish Peroxidase) conjugate in an anti-CNOT7 or anti-LAIR-1 antibody pre-coated ELISA plate for an hour at 37 °C. After the incubation period, wells were decanted and washed five times manually using a pre-diluted wash buffer provided by the manufacturer. A substrate for HRP enzyme was incubated for an hour at 37 °C followed by the stop solution. The color intensity produced was measured spectrophotometrically at 450 nm in a microplate reader (Awareness Technology, Palm City, FL, USA). A standard curve was plotted relating the color intensity (Optical Density (OD)) to the concentration of standards. The CNOT7 or LAIR-1 concentration in each sample was interpolated from this standard curve (Supplementary File Figure S1).

#### 5.3.2. Insulin ELISA

Serum insulin assay was performed using Hyperion Inc., Miami, FL, USA, ELISA kit. This solid-phase ELISA utilizes two anti-insulin antibodies to form a sandwich complex. Standards and serum samples were incubated in antibody-coated wells, followed by the addition of HRP-conjugated anti-insulin antibody. After incubation at room temperature and washing, TMB substrate (3,3′,5,5′-Tetramethylbenzidine substrate) was added, leading to color development. The reaction was stopped and absorbance was measured at 450 nm. Insulin concentrations were interpolated from a standard curve, with the normal adult range being 0–25 mU/L.

#### 5.3.3. CNOT7 IHC Staining Protocol

CNOT7 IHC staining protocol was performed using paraffin sections mounted on positively charged slides using avidin–biotin–peroxidase complex (ABC) technique [57]. Sections from each group were treated with CNOT7 (M01) (ABNOVA, Cat# H00029883-M01, Monoclonal antibody, Clone 2F6) at 1:25 dilution before being exposed to the chemicals necessary for the ABC method (Vectastain ABC-HRP kit, Vector laboratories, Newark, CA, USA). To detect antigen–antibody complexes, marker expression was tagged with peroxidase and colored with diaminobenzidine (DAB, Sigma, cat# D5637, St. Louis, MO, USA). Negative controls were included using non-immune serum in place of the primary or secondary antibodies. A Leica microscope model CH9435 Hee56rbrugg manufactured by Leica Microsystems (Heerbrugg, Switzerland) was used to evaluate IHC-stained sections.

#### 5.3.4. Quantitative Evaluation of CNOT7 IHC Results “Area %”

In each serial section of the analyzed patients’ groups, six high-power fields (×400) with positive brown immunostaining were chosen for examination. The area % of CNOT7-stained sections was calculated using the Leica QWin 500 image analyzer computer system (UK). This image analyzer utilized a Leica microscope, a colored video camera, a colored monitor, and a hard disc of a Leica IBM personal computer linked to the microscope and managed by Leica QWin 500 software.

Area % data were statistically reported in terms of mean and standard error of mean (mean ± SEM) for eight images from four non-metastatic samples and four metastatic samples.

### 5.4. Statistical Analysis

Data collected were recorded and analyzed using the Statistical Package for Social Science software (SPSS, Version 17, Chicago, IL, USA). Qualitative data were presented as frequencies (*n*) and percentages (%). Test for quantitative data normality was performed using the Shapiro–Wilk calculator. Normally distributed variables were represented as mean ± SEM and analyzed using two-sample independent Student’s *t*-test for comparison of two groups. Data that were not normally distributed were presented as medians (interquartile ranges as first to third quartiles or 25th–75th quartiles) and analyzed using the Mann–Whitney (U) test.

ROC curve was calculated to detect the best cut-off points for categorizing CNOT7 and LAIR-1 serum levels into low- and high-expression subgroups.

The Chi-square test was used to evaluate the association between CNOT7 serum levels and clinicopathological parameters. Pearson correlation test was used to assess the association between parametric variables. Point-biserial correlations were used to determine the correlation between parameters when one of them was a dichotomous variable.

The level of significance was set at *p* < 0.05 and the confidence level (CI) interval at 95% and 5%.

### 5.5. In Silico Bioinformatics Analysis

#### 5.5.1. Breast Cancer Gene Expression Miner v5.0 (bc-GenExMiner v5.0) Updated on 28 June 2023

The Breast Cancer Gene Expression Miner v5.0 (bc-GenExMiner v5.0) http://bcgenex.ico.unicancer.fr/BC-GEM/GEM-Accueil.php?js=1 accessed on 8 May 2023 [58] was used to explore the correlation between CNOT7 and LAIR-1.

#### 5.5.2. Function Module of LinkedOmics

The function module of LinkedOmics was employed to perform analysis of Gene Ontology biological process (GO_BP) along with gene set enrichment analysis (GSEA) http://www.linkedomics.org/ was accessed on 23 April 2023. [59].

#### 5.5.3. Gene–Gene Interactions and Pathways

Gene–gene interactions and pathways were generated using in silico curated databases (DBs) and text mining for target genes using University of California Santa Cruz (UCSC) [60] Genomics Institute Genome Browser http://genome.ucsc.edu/index.html. Interactions between LAIR-1 and CNOT7, along with known inhibitor drugs from DrugBank, were retrieved. This DB was accessed on 20 June 2024.

#### 5.5.4. Protein–Protein Interactions (PPIs)

Protein–protein interactions were generated using the Search Tool for the Retrieval of Interacting Genes/Proteins (STRING) database version 11.5 https://string-db.org/, which predicts interactions based on relevant experimental data, sequencing results, and literature from >1100 fully sequenced organisms [61]. This DB was accessed on 20 January 2023 to explore LAIR-1-interacting proteins.

#### 5.5.5. Exploring Gene Expression Patterns Across Normal and Tumor Tissues

To investigate gene expression patterns across normal and tumor tissues, we utilized the GENT2 database (http://gent2.appex.kr/gent2/) that was accessed 18 May 2024. This platform allows for comprehensive analysis of gene expression and survival data, providing insights into the differential expression of genes between normal and cancerous tissues.

### 5.6. Molecular Docking

#### 5.6.1. Preparation of the Protein Structures of LAIR-1 and CNOT7

To conduct protein–ligand docking, we obtained the crystal structure of Human LAIR-1 (PDB ID: 3RP1) from the Protein Data Bank (PDB) (https://www.rcsb.org/structure/3RP1, accessed on 23 July 2025). After loading the structures into the Molecular Operating Environment (MOE) (https://www.chemcomp.com/Products.htm, accessed on 23 July 2025), an Integrated Computer-Aided Molecular Design Platform, we prepared the structures using the default parameters in the ‘QuickPrep Panel’. This preparation included the removal of water molecules, addition of hydrogen atoms to the protein structure, adjustment of protonation states, and energy minimization of the protein structures using the Amber ETH10 force field.

Because these crystal structures were not co-crystallized with any ligands, we used the MOE SiteFinder tool, which is equipped for binding-site analysis, to predict possible binding sites. These binding pockets were ranked based on their propensity for ligand binding (PLB).

CNOT7 is a receptor without an available PDB co-crystallized structure. Therefore, we utilized the MOE SiteFinder tool to identify potential binding sites. Guided by previous literature, we identified several essential amino acid residues involved in ligand–CNOT7 protein interactions, such as Glu247, Tyr260, and Glu278, as relatively significant [62,63].

#### 5.6.2. Ligand Dataset Curation

The ‘builder program’ implemented in the MOE was used to generate the 3D structures of ligands included in the study: vitamin E, colchicine, prodigiosin, riboflavin, telmisartan, and pioglitazone. Pronotation state at physiological condition was considered.

#### 5.6.3. Docking Simulations

Docking of the obtained conformations of the six ligands was carried out using the docking module of the MOE. Initial docking of the molecules in the active sites used the ‘Triangle Matcher’ placement method and ‘London dG’ scoring function. Further refinement of docking poses was achieved using ‘GBVI/WSA dG’ scoring method. The poses with minimum energy were used for visualization of the binding interactions as well as occupancy of the binding site of the receptors.

## 6. Summary and Conclusions

Overall, this study highlights the importance of CNOT7 in BC progression. Further investigations into the possible roles of CNOT7 in metastatic progression may reveal additional important insights into tumor autonomous metastatic mechanisms. Whether the interaction between CNOT7 and the TIME as well as LAIR-1 is conserved and whether the reduced expression of LAIR-1 and the abundance of CNOT7 are mechanistically linked remain to be investigated via additional mechanistic studies.

## 7. Therapeutic Implication(s)

Overall, the docking studies suggest that riboflavin, pioglitazone, telmisartan, and colchicine may be potent modulators of CNOT7 and LAIR-1 receptors due to their strong interactions with key residues. These findings highlight the potential of these ligands as therapeutic agents, particularly in conditions where the modulation of CNOT7 or LAIR-1 activity is beneficial. Therefore, it is recommended that further experimental studies are performed to validate these interactions and explore their biological implications in detail.

## 8. Recommendations and Future Perspectives

Future research should address structure–activity relationships (SARs) to characterize cellular target inhibitors or mimetics/agonists. Second, future research should test blood granzyme B, insulin receptor, insulin-like growth factor, and apelin, as well as lipocalins, in relation to CNOT7 as accompanying risk factors or prognosis markers of diabetes or obesity in metastatic BC [64,65,66,67,68,69]. Moreover, DNA damage and hypoxia-inducible factor should be examined in relation to CNOT7 (an untouched area of research) [70,71,72]. Third, future works should develop a cancer vaccine against CNOT7 targets via a reverse vaccinology approach using our previous immuno/bioinformatics approach [73].

## 9. Limitation(s), Possible Research Gap(s)

Future research should target other CCR4-NOT complex subunits in other cancer types and examine the non-coding RNA (ncRNA) signature for CNOT7 induction and its relation to multidrug resistance in metastatic BC treatment. This is a potential option for cancer treatment, based on ncRNA, and a step forward toward precision medicine (PM) that constitutes a promising research gap to be addressed as a sustainability plan.

## Figures and Tables

**Table 1:** Study participants’ demographic and clinical characteristics in the BC non-metastatic (*n* = 46) vs. metastatic BC (*n* = 44) groups.

	Group (n)		Statistics
Characteristic (Unit)	Non-Metastatic (46)	Metastatic (44)	*p*
**Age (years) (n,%)**			ꭓ^2^ = 2.820 *NS*
**≤50**	(18, 39.1%)	(25, 56.8%)	
**>50**	(28, 60.9%)	(19, 43.2%)	
**BMI (kg/m^2^)**			ꭓ^2^ = 6.673 *p* = 0.036 *
**Normal (18.5–24.9) (n, %)**	(1, 2.2%)	(2, 4.5%)	
**Overweight (25–29.9) (n, %)**	(5, 10.9%)	(14, 31.8%)	
**Obese (≥30) (n, %)**	(40, 87.0%)	(28, 63.6%)	
**Diabetic status**			ꭓ^2^ = 2.883 *NS*
**Yes (n, %)**	(20, 43.5%)	(27, 61.4%)	
**No (n, %)**	(26, 56.5%)	(17, 38.6%)	
**No. of pregnancies**			ꭓ^2^ = 1.558 *NS*
**≤2 (n, %)**	(17, 37.0%)	(22, 50.0%)	
**>2 (n, %)**	(29, 63.0%)	(22, 50.0%)	
**Menopausal status**			ꭓ^2^ = 0.552 *NS*
**Pre (n, %)**	(21, 45.7%)	(24, 54.5%)	
**Post (n, %)**	(24, 52.2%)	(20, 45.5%)	
**BC Family History**			ꭓ^2^ = 0.829 *NS*
**Yes (n, %)**	(17, 37.0%)	(12, 27.3%)	
**No (n, %)**	(29, 63.0%)	(31, 70.5%)	
**Histological BC subtype**			ꭓ^2^ = 2.007 *NS*
**IDC (n, %)**	(43, 93.5%)	(37, 84.1%)	
**Other subtypes (n, %)**	(3, 6.5%)	(7, 15.9%)	
**BC molecular subtype**			
**Luminal A (n, %)**	(34, 73.9%)	(17, 38.6%)	ꭓ^2^ = 15.830 *p* = 0.001 *
**Luminal B (n, %)**	(9, 19.6%)	(18, 40.9%)	
**HER-2 overexpression (n, %)**	(3, 6.5%)	(2, 4.5%)	
**TNBC (n, %)**	(0, 0%)	(7, 15.9%)	
**ER status**			ꭓ^2^ = 4.779 *p* = 0.029 *
**Positive (n, %)**	(43, 93.5%)	(34, 77.3%)	
**Negative (n, %)**	(3, 6.5%)	(10, 22.7%)	
**PR status**			ꭓ^2^ = 1.213 *NS*
**Positive (n, %)**	(37, 80.4%)	(31, 70.5%)	
**Negative (n, %)**	(9, 19.6%)	(13, 29.5%)	
**HER-2/neu status**			ꭓ^2^ = 1.176 *NS*
**Positive (n, %) High**	(12, 26.1%)	(9, 20.5%)	
**Negative (n, %) Low**	(12, 26.1%)	(16, 36.4%)	
**None**	(22, 47.8%)	(19, 43.2%)	
**Tumor size (cm)**			ꭓ^2^ = 2.402 NS
**<2 (n, %)**	(6, 13.0%)	(2, 4.5%)	
**2–5 (n, %)**	(31, 67.4%)	(30, 68.2%)	
**>5 (n, %)**	(9, 19.6%)	(12, 27.3%)	
**LN involvement**			ꭓ^2^ = 13.074 *p* = 0.004 *
**None (n, %)**	(19, 41.3%)	(7, 15.9%)	
**1–3 (n, %)**	(13, 28.3%)	(11, 25.0%)	
**4–9 (n, %)**	(5, 10.9%)	(18, 40.9%)	
**≥10 (n, %)**	(9, 19.6%)	(8, 18.2%)	
**TNM stage**			ꭓ^2^ = 40.927 *p* = 0.001 *
**I-II (early stage) (n, %)**	(29, 63.0%)	(0, 20.0%)	
**III-IV (late stage) (n, %)**	(17, 37.0%)	(44, 100.0%)	
**Grade (Bloom–Richardson scale)**			ꭓ^2^ =7.396 *p* =0.025 *
**I (n, %)**	(2, 4.3%)	(0, 0%)	
**II (n, %)**	(30, 65.2%)	(39, 88.6%)	
**III (n,%)**	(14, 30.4%)	(5, 11.4%)	
**Ki-67 status**			ꭓ^2^ = 1.472 NS
**High (>14%) (n, %)**	(21, 45.7%)	(24, 54.5%)	
**Low (≤14%) (n, %)**	(12, 26.1%)	(7, 15.9%)	
**NA (n, %)**	(13, 28.3%)	(13, 29.5%)	
**Therapy**			
**Neoadjuvant chemotherapy**			ꭓ^2^ = 26.058 *p* < 0.001 *
**Yes (n, %)**	(7, 15.2%)	(30, 68.2%)	
**No (n, %)**	(39, 84.8%)	(14, 31.8%)	
**Adjuvant chemotherapy**			ꭓ^2^ = 1.574 NS
**Yes (n, %)**	(32, 69.6%)	(25, 56.8%)	
**No (n, %)**	(14, 30.4%)	(19, 43.2%)	
**Endocrine therapy**			ꭓ^2^ = 0.005 NS
**Yes (n, %)**	(30, 65.2%)	(29, 65.9%)	
**No (n, %)**	(16, 34.8%)	(15, 34.1%)	
**Mastectomy**			ꭓ^2^ = 0.356 NS
**Yes (n, %)**	(32, 69.6%)	(28, 63.6%)	
**No (n, %)**	(14, 30.4%)	(16, 36.4%)	
**Insulin (uIU/mL) ^#^**	30.865 (24.837–49.484)	30.578 (23.027–45.919)	NS

Data are presented as (n, %) or ^#^ median (interquartile range (1st–3rd quartile)) for non-parametric data. Statistics were computed using Chi-square test (dichotomous parameters). * Statistical significance is set at *p* < 0.05. [BMI—body mass index; BC—breast cancer; ki-67—marker of proliferation index Ki-67; LN—lymph node; IDC—invasive ductal carcinoma; HER-2—human epidermal growth factor receptor 2; TNBC—triple-negative breast cancer; ER—estrogen receptor; PR—progesterone receptor; TNM—tumor–node–metastasis; NA—not applicable/not available; NS—non-significant].

**Table 2:** Association between LAIR-1 and CNOT7 serum levels (low and high) and clinicopathological variables in the BC patients’ cohort (n = 90).

Characteristic (Unit)	LAIR-1 Serum Levels (≤61.01)		Statistics *p*	CNOT7 Serum Levels (≤3.62)		Statistics *p*
	Low	High		Low	High	
**Age (years)**			ꭓ^2^ = 0.062 *NS*			ꭓ^2^ = 3.607 *NS*
**≤50 (n, %)**	(19, 46.3%)	(24, 49.0%)		(26, 57.8%)	(17, 37.8%)	
**>50 (n, %)**	(22, 53.7%)	(25, 51.0%)		(19, 42.2%)	(28, 62.2%)	
**BMI (kg/m^2^)**			ꭓ^2^ = 0.630 *NS*			ꭓ^2^ = 6.067 *p* = 0.048 *
**Normal (18.5–24.9) (n, %)**	(2, 4.9%)	(1, 2.0%)		(1, 2.2%)	(2, 4.4%)	
**Overweight (25–29.9) (n, %)**	(8, 19.5%)	(11, 22.4%)		(5, 11.1%)	(14, 31.1%)	
**Obese (≥30) (n, %)**	(31, 75.6%)	(37, 75.5%)		(39, 86.7%)	(29, 64.4%)	
**Diabetic status**			ꭓ^2^ = 7.795 *p* = 0.005 *			ꭓ^2^ = 3.607 *NS*
**Yes (n, %)**	(28, 68.3%)	(19, 38.8%)		(19, 42.2%)	(28, 62.2%)	
**No (n, %)**	(13, 31.7%)	(30, 61.2%)		(26, 57.8%)	(17, 37.8%)	
**No. of pregnancies**			ꭓ^2^ = 0.010 *NS*			ꭓ^2^ = 0.045 *NS*
**≤2 (n, %)**	(18, 43.9%)	(21, 42.9%)		(20, 44.4%)	(19, 42.2%)	
**>2 (n, %)**	(23, 56.1%)	(28, 57.1%)		(25, 55.6%)	(26, 57.8%)	
**Menopausal status**			ꭓ^2^= 0.109 *NS*			ꭓ^2^= 12.231 *p* = 0.0005 *
**Pre (n, %)**	(21, 51.2%)	(24, 49.0%)		(31, 68.9%)	(14, 31.1%)	
**Post (n, %)**	(19, 46.3%)	(25, 51.0%)		(14, 31.1%)	(30, 66.7%)	
**BC Family History**			ꭓ^2^ = 1.902 *NS*			ꭓ^2^ = 0.023 *NS*
**Yes (n, %)**	(10, 24.4%)	(19, 38.8%)		(15, 33.3%)	(14, 31.1%)	
**No (n, %)**	(30, 73.2%)	(30, 61.2%)		(30, 66.7%)	(30, 66.7%)	
**Histological BC subtype**			ꭓ^2^ = 0.090 *NS*			ꭓ^2^ = 1.80 *NS*
**IDC (n, %)**	(36, 87.8%)	(44, 89.9%)		(38, 84.4%)	(42, 93.3%)	
**Other subtypes (n, %)**	(5, 12.2%)	(5, 10.2%)		(7, 15.6%)	(3, 6.7%)	
**BC molecular subtype**			ꭓ^2^ = 5.812 *NS*			ꭓ^2^ = 8.077 *p* = 0.044 *
**Luminal A (n, %)**	(20, 48.8%)	(31, 63.3%)		(31, 68.9%)	(20, 44.4%)	
**Luminal B (n, %)**	(12, 29.3%)	(15, 30.6%)		(12, 26.7%)	(15, 33.3%)	
**HER-2 overexpression (n, %)**	(3, 7.3%)	(2, 4.1%)		(1, 2.2%)	(4, 8.9%)	
**TNBC (n, %)**	(6, 14.6%)	(1, 2.0%)		(1, 2.2%)	(6, 13.3%)	
**BC molecular subtype combined**			ꭓ^2^ = 5.618 *NS*			ꭓ^2^ = 6.192 *p* = 0.045 *
**Luminal (n, %)**	(32, 78.0%)	(46, 93.9%)		(43, 95.6%)	(35, 77.8%)	
**HER-2 overexpression (n, %)**	(3, 7.3%)	(2, 4.1%)		(1, 2.2%)	(4, 8.9%)	
**TNBC (n, %)**	(6, 14.6%)	(1, 2.0%)		(1, 2.2%)	(6, 13.3%)	
**ER status**			ꭓ^2^ = 6.028 *p* = 0.014 *			ꭓ^2^ = 7.283 *p* = 0.007 *
**Positive (n, %)**	(31, 75.6%)	(46, 93.9%)		(43, 95.6%)	(34, 75.6%)	
**Negative (n, %)**	(10, 24.4%)	(3, 6.1%)		(2, 4.4%)	(11, 24.4%)	
**PR status**			ꭓ^2^ = 3.838 *p* = 0.05 *			ꭓ^2^ = 6.016 *p* = 0.014 *
**Positive (n, %)**	(27, 65.9%)	(41, 83.7%)		(39, 86.7%)	(29, 64.4%)	
**Negative (n, %)**	(14, 34.1%)	(8, 16.3%)		(6, 13.3%)	(16, 35.6%)	
**HER-2/neu status**			ꭓ^2^ = 0.652 *NS*			ꭓ^2^ = 1.553 *NS*
**Positive (n, %)**	(8, 19.5%)	(13, 26.5%)		(8, 17.8%)	(13, 28.9%)	
**Negative (n, %)**	(33, 80.5%)	(36, 73.5%)		(37, 82.2%)	(32, 71.1%)	
**Tumor size (cm)**			ꭓ^2^ = 0.645 *NS*			ꭓ^2^ = 0.064 *NS*
**<2 (n, %)**	(3, 7.3%)	(5, 10.2%)		(4, 8.9%)	(4, 8.9%)	
**2–5 (n, %)**	(27, 65.9%)	(34, 69.4%)		(31, 68.9%)	(30, 66.7%)	
**>5 (n, %)**	(11, 26.8%)	(10, 20.4%)		(10, 22.2%)	(11, 24.4%)	
**LN involvement**			ꭓ^2^ = 2.980, *NS*			ꭓ^2^ = 9.795 *p* = 0.020 *
**None (n, %)**	(10, 24.4%)	(16, 32.7%)		(15, 33.3%)	(11, 24.4%)	
**1–3 (n, %)**	(10, 24.4%)	(14, 28.6%)		(17, 37.8%)	(7, 15.6%)	
**4–9 (n, %)**	(14, 34.1%)	(9, 18.4%)		(8, 17.8%)	(15, 33.3%)	
**≥10 (n, %)**	(7, 17.1%)	(10, 20.4%)		(5, 11.1%)	(12, 26.7%)	
**TNM stage**			ꭓ^2^ = 10.667 *p* = 0.001 *			ꭓ^2^ = 6.156 *p* = 0.013 *
**I-II (early stage) (n, %)**	(6, 14.6%)	(23, 46.9%)		(20, 44.4%)	(9, 20.0%)	
**III-IV (late stage) (n, %)**	(35, 85.4%)	(26, 53.1%)		(25, 55.6%)	(36, 80.0%)	
**Grade (Bloom–Richardson scale)**			ꭓ^2^ = 4.393 *NS*			ꭓ^2^ = 2.067 *NS*
**I (n, %)**	(0, 0%)	(2, 4.1%)		(2, 4.4%)	(0, 0%)	
**II (n, %)**	(29, 70.7%)	(40, 81.6%)		(34, 75.6%)	(35, 77.8%)	
**III (n, %)**	(12, 29.3%)	(7, 14.3%)		(9, 20.0%)	(10, 22.2%)	
**Ki-67 status**			ꭓ^2^ = 2.327 *NS*			ꭓ^2^ = 2.178 *NS*
**High (>14%) (n, %)**	(19, 46.3%)	(26, 53.1%)		(11, 24.4%)	(8, 17.8%)	
**Low (≤14%) (n, %)**	(7, 17.1%)	(12, 24.5%)		(19, 42.2%)	(26, 57.8%)	
**NA (n, %)**	(15, 36.6%)	(11, 22.4%)		(15, 33.3%)	(11, 24.4%)	
**Therapy**						
**Neoadjuvant chemotherapy**			ꭓ^2^ = 3.178 *NS*			ꭓ^2^ = 5.553 *p* = 0.018 *
**Yes (n, %)**	(21, 51.2%)	(16, 32.7%)		(13, 28.9%)	(24, 53.3%)	
**No (n, %)**	(20, 48.8%)	(33, 67.3%)		(32, 71.1%)	(21, 46.7%)	
**Adjuvant chemotherapy**			ꭓ^2^ = 0.746 *NS*			ꭓ^2^ = 8.086 *p* = 0.004 *
**Yes (n, %)**	(24, 58.5%)	(33, 67.3%)		(35, 77.8%)	(22, 48.9%)	
**No (n, %)**	(17, 41.5%)	(16, 32.7%)		(10, 22.2%)	(23, 51.1%)	
**Endocrine therapy**			ꭓ^2^ = 0.003 *NS*			ꭓ^2^ = 1.23 *NS*
**Yes (n, %)**	(27, 65.9%)	(32, 65.3%)		(32, 71.1%)	(27, 60.0%)	
**No (n, %)**	(14, 34.1%)	(17, 34.7%)		(13, 28.9%)	(18, 40.0%)	
**Mastectomy**			ꭓ^2^ = 1.434 *NS*			ꭓ^2^ = 3.200 *NS*
**Yes (n, %)**	(30, 73.2%)	(30, 61.2%)		(26, 57.8%)	(34, 75.6%)	
**No (n, %)**	(11, 26.8)	(11, 38.8%)		(19, 42.2%)	(11, 24.4%)	

Data are presented as (n, %). Statistics computed using Chi-square test (dichotomous parameters). * Statistical significance is set at *p* < 0.05. [BMI—body mass index; BC—breast cancer; ki-67—marker of proliferation index Ki-67; LN—lymph node; IDC—invasive ductal carcinoma; HER-2—human epidermal growth factor receptor 2; TNBC—triple-negative breast cancer; ER—estrogen receptor; PR—progesterone receptor; TNM—tumor–node–metastasis; NA—not applicable/not available; NS—non-significant].

**Table 3:** Correlation of CNOT7 serum levels and BC patients’ characteristics (n = 90).

*Patient Characteristic*	*CNOT7 r, p*
*Age #*	−0.034, NS
*BMI #*	0.065, NS
*Diabetic status*	0.147, NS
*No. of pregnancies*	−0.079, NS
***Menopausal status***	0.292, 0.006 *
*BC family history*	−0.162, NS
*Histological subtype*	−0.198, NS
*Molecular subtype*	−0.063, NS
***ER status***	−0.209, 0.048 *
*PR status*	−0.166, NS
*HER-2 status*	0.105, NS
*Tumor size*	0.029, NS
***LN involvement***	0.226, 0.032 *
***TNM stage***	0.256, 0.015 *
*Grade (Bloom–Richardson scale)*	−0.063, NS

# Pearson correlation coefficient was used to measure the degree of association between two continuous parametric variables (age or BMI). Point-biserial correlation was used to measure the association that exists between two variables, one continuous and the other dichotomous (used for the rest). * Significant statistical difference; *p*-value less than 0.05. [BMI—body mass index; BC—breast cancer; LN—lymph node; HER-2—human epidermal growth factor receptor 2; ER—estrogen receptor; PR—progesterone receptor, TNM—tumor–node–metastasis; NS—non-significant].

## Data Availability

Data are contained within the article or Supplementary Materials.

## Supplementary Materials

The following supporting information can be downloaded at: https://www.mdpi.com/article/10.3390/ijms26157141/s1.

## Abbreviations

AFG3L2	AFG3-like AAA ATPase 2
AGO	Argonaute RISC catalytic component
ALDH7A1	Aldehyde dehydrogenase 7 family, member A1
APP	Amyloid beta (A4) precursor protein
BAG3	BCL2-associated athanogene 3
BC	Breast cancer
BTG	B-cell translocation gene
CAPZA	Capping protein (actin filament) muscle Z-line, alpha
CDK	Cyclin-dependent kinase
CKB	Creatine Kinase B
EIF	Eukaryotic translation initiation factor
EPCAM	Epithelial cell adhesion molecule
GO	Gene Ontology
GSEA	Gene set enrichment analysis
HCC	Hepatocellular carcinoma
IFN-γ	Interferon-γ
INF	Interferon
LAIR-1	Leukocyte-associated immunoglobulin-like receptor-1
NK	Natural killer
NKKB1	Nuclear Factor Kappa B Subunit 1
PABPC	Poly(A) binding protein cytoplasmic like
PI3K/AKT/mTOR	Phosphoinositide 3 kinase (PI3K)/AKT/mammalian (or mechanistic) target of rapamycin (mTOR)
PTK	Protein tyrosine kinase
PTP	Protein tyrosine phosphatase
PTPN	Protein tyrosine phosphatase non-receptor
RELA	V-rel avian reticuloendotheliosis viral oncogene homolog A
STAT1	Signal transducer and activator of transcription 1
TGF-β1	Transforming growth factor-β1
TIME	Tumor immunosuppressive microenvironment
TME	Tumor microenvironment
UBC	Ubiquitin C
