# Supplementary material for: CCR4-NOT Transcription Complex Subunit 7 (CNOT7) Protein and Leukocyte-Associated Immunoglobulin-like Receptor-1 in Breast Cancer Progression: Clinical Mechanistic Insights and In Silico Therapeutic Potential
Source: Int J Mol Sci. 2025 Jul 24;26(15):7141. doi: 10.3390/ijms26157141 (PMC12346410; doi:10.3390/ijms26157141)
Supplement: Supplementary file 1 [file ijms-26-07141-s001.zip › ijms-3710557-supplementary.pdf]

CCR4-NOT Transcription Complex Subunit 7 (**CNOT7)** protein

and **Leukocyte-associated immunoglobulin-like receptor 1 in Breast Cancer Progression: Clinical Mechanistic-Insights and In-silico Therapeutic Potential**

A

**CNOT7 (ng/ml)**	**Absorbance (450 nm)**
0	2.887
0.5	2.173
1	1.649
5	0.822
10	0.544

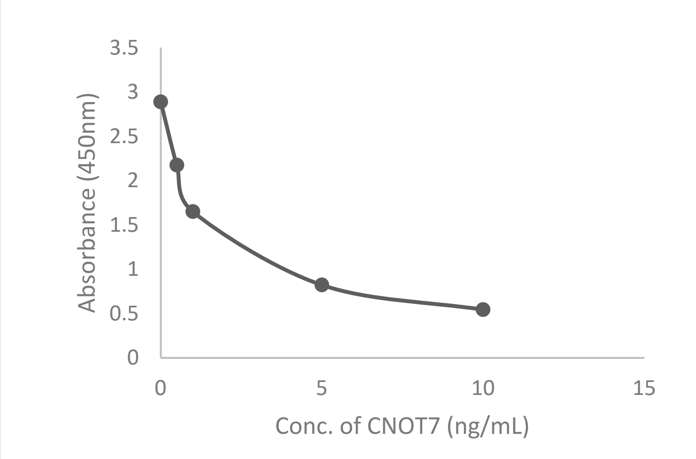

B

**LAIR-1 (nmol/L)**	**Absorbance (450 nm)**
29.856	0.392
55.374	0.615
142.17	1.223
221.728	1.691
477.448	2.958

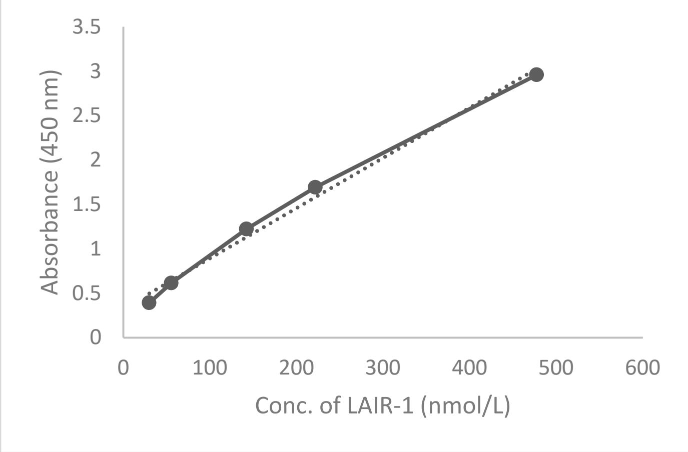

C

Insulin (µIU/mL)	Absorbance (450 nm)
0	0.075
5	0.148
25	0.497
50	0.955
100	1.716
200	3.039

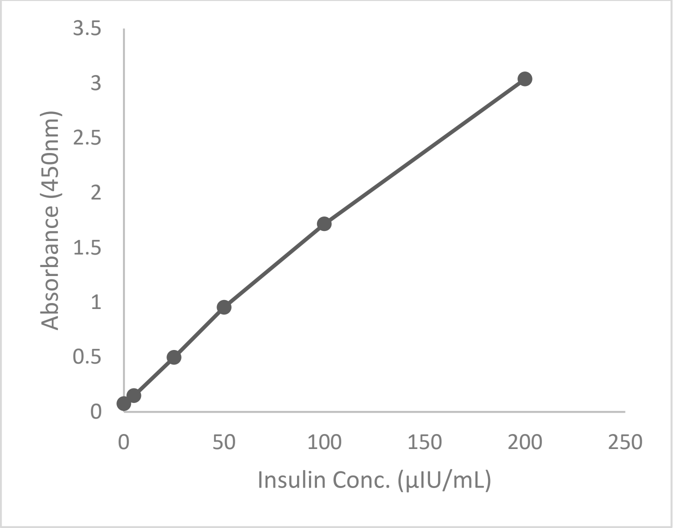

1 **Figure S1. ELISA Standard curve for A) CNOT7 expected values and sensitivity, B)**
2 **LAIR-1 expected values and sensitivity, and C) s.Insulin expected values and sensitivity**

Non-Metastatic

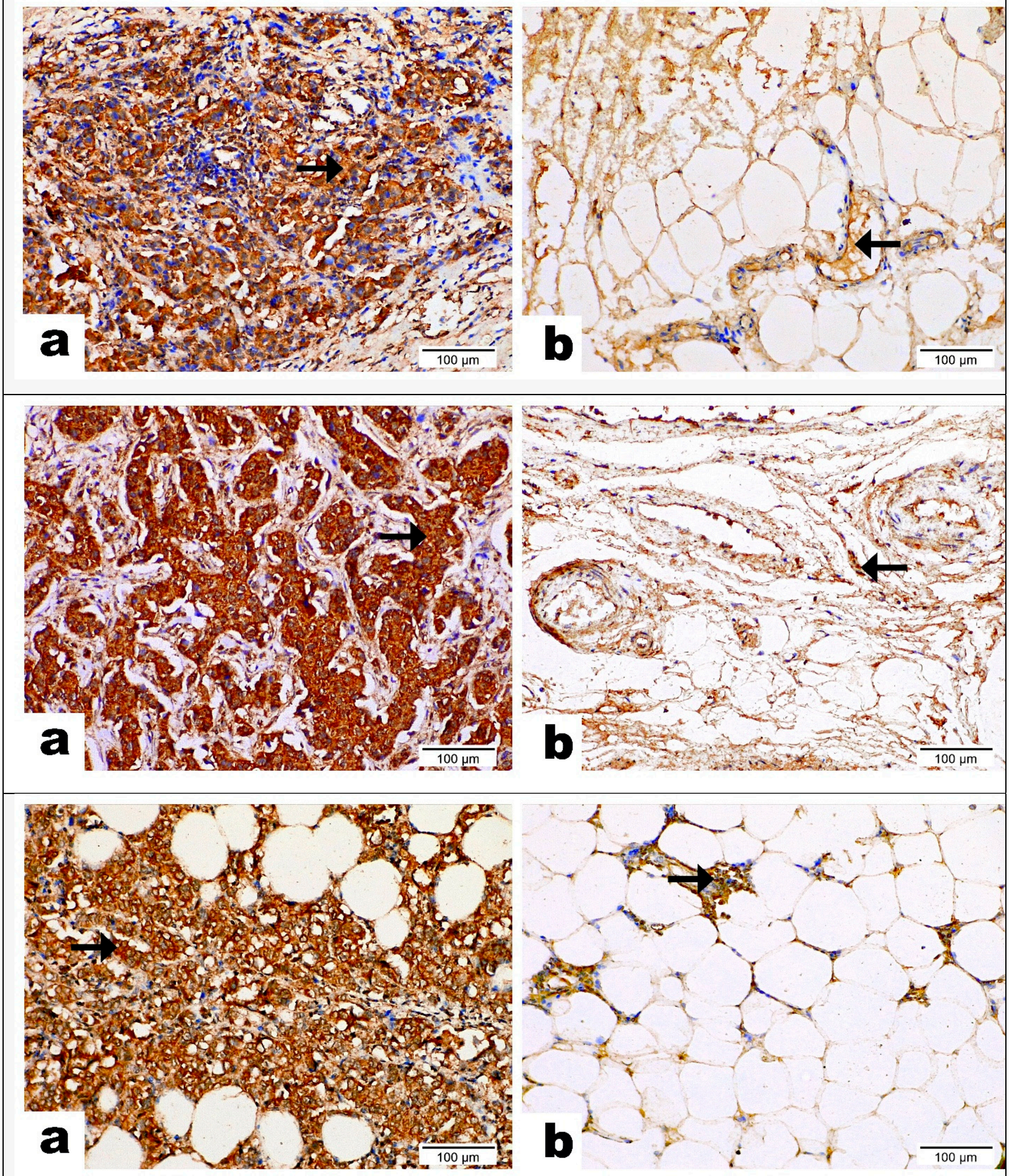

Metastatic

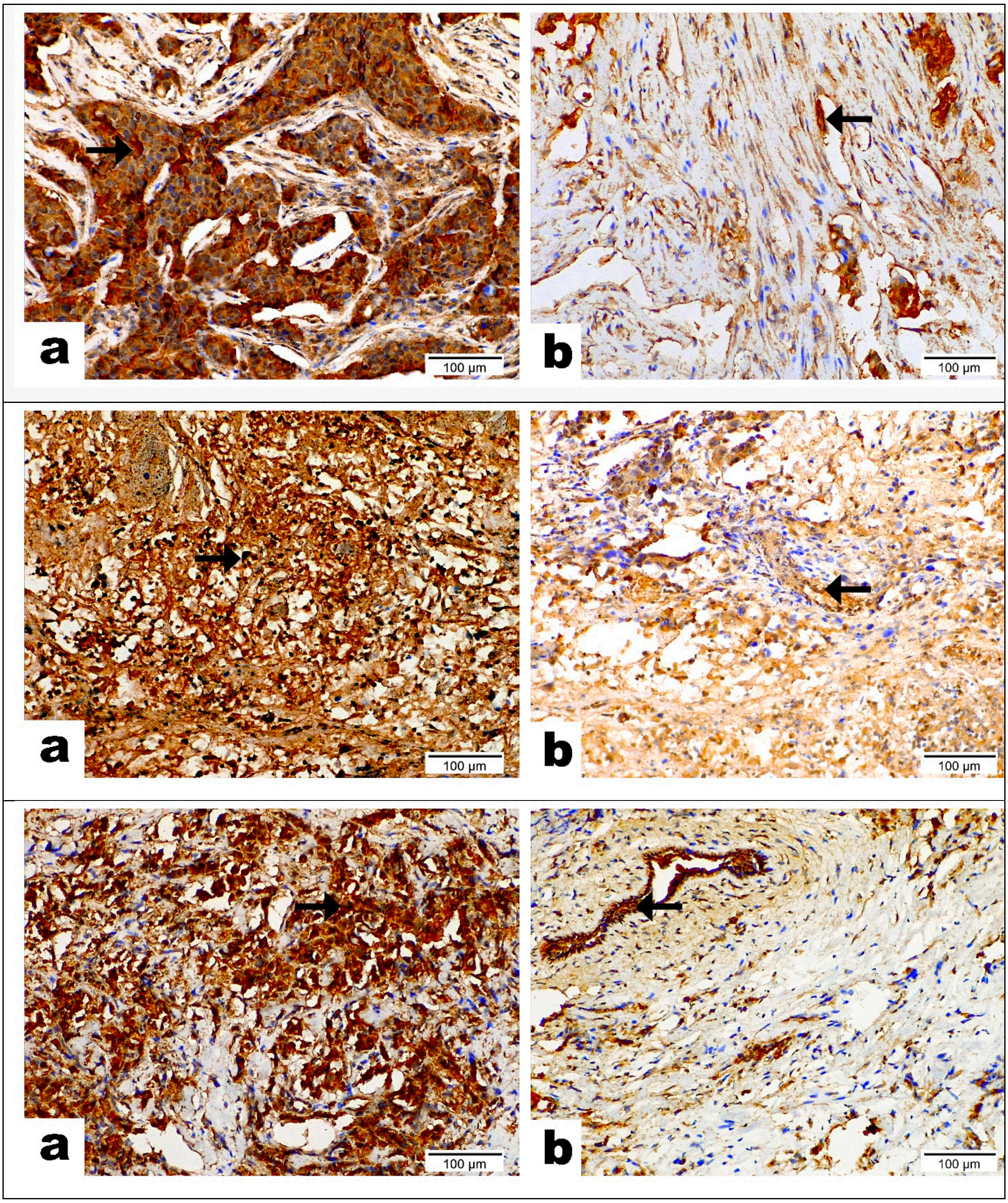

3 **Figure S2. IHC analysis of CNOT7 expression in BC tissue (n=4) (a) and its adjacent healthy**
4 **breast tissues (b) in metastatic BC and non-metastatic BC groups.** Cells with yellow-brown
5 staining are immune positive and the arrows denote the intracellular localization of CNOT7.

(Sections 100 um, Magnification x400). **CNOT7 is over-expressed in the metastatic BC tissues when compared to the non-metastatic BC tissues.**

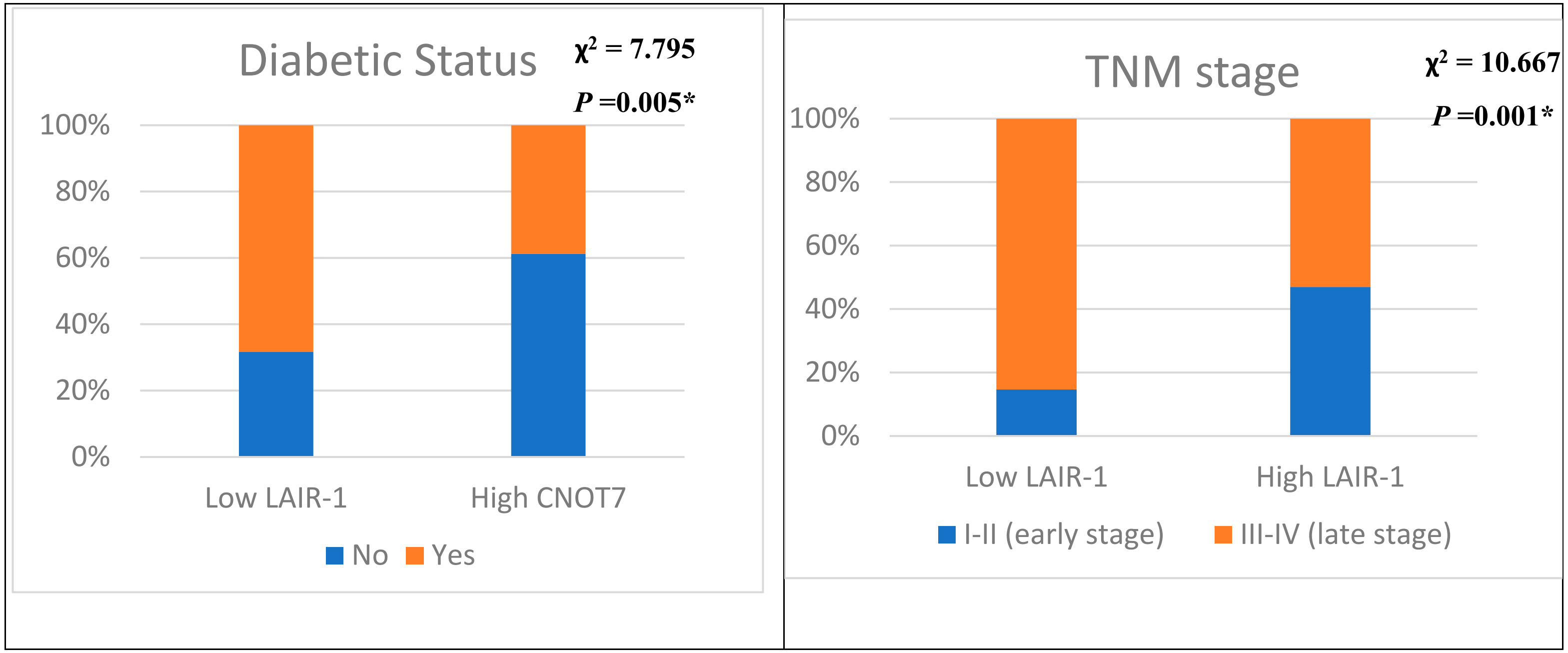
Diabetic Status
χ² = 7.795
P =0.005*
100%
80%
60%
40%
20%
0%
Low LAIR-1
High CNOT7
No
Yes
TNM stage
χ² = 10.667
P =0.001*
100%
80%
60%
40%
20%
0%
Low LAIR-1
High LAIR-1
I-II (early stage)
III-IV (late stage)

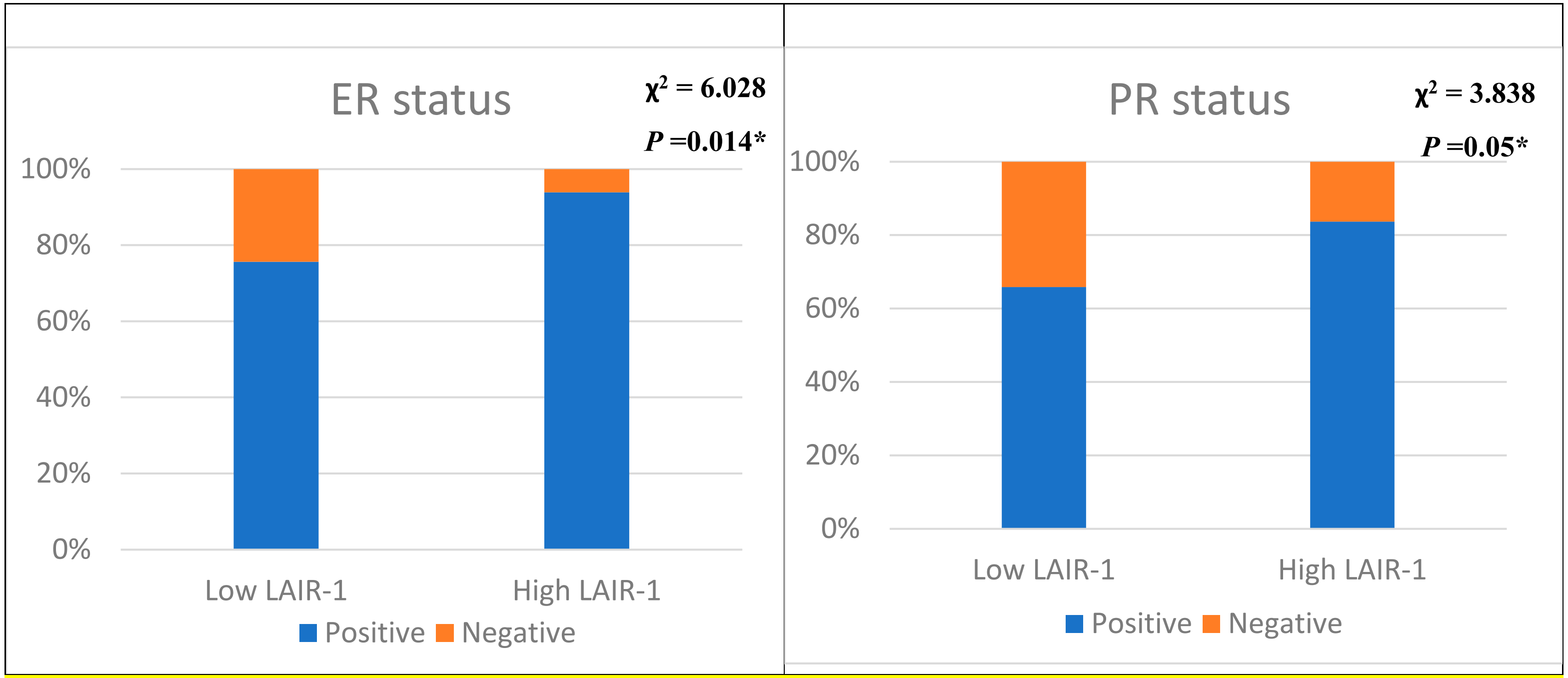

Figure S3. Significant associations between LAIR-1 serum levels (low and high) and clinicopathological variables in BC patients (n=90). Data are presented as count (n) and percentage (%). Statistical significance was determined using the Chi-square test (for dichotomous parameters), with significance set at $P<0.05$.

Significant associations were observed for Diabetic status (P=0.005), ER status (P=0.014), PR status (P=0.05), and TNM stage (P=0.001). Non-significant (NS) associations are not included in this figure, focusing only on the relationships identified as statistically significant in Table 2. [BMI, body mass index; BC, breast cancer; LN, lymph node; IDC, invasive

ductal carcinoma; HER-2, human epidermal growth factor receptor 2; TNBC, triple negative breast cancer; ER, estrogen receptor; PR, progesterone receptor; TNM, tumor node metastasis.]

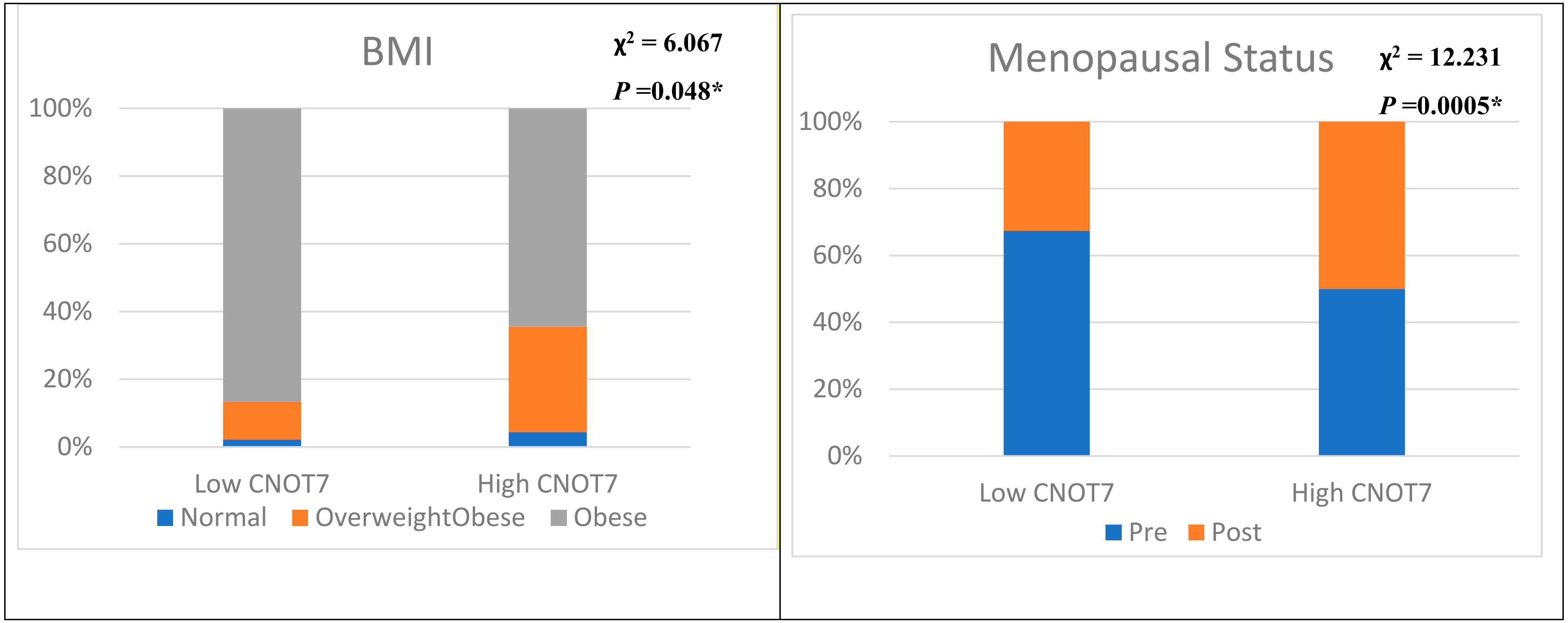
BMI
χ2 = 6.067
P =0.048*
100%
80%
60%
40%
20%
0%
Low CNOT7
High CNOT7
Normal
OverweightObese
Obese
Menopausal Status
χ2 = 12.231
P =0.0005*
100%
80%
60%
40%
20%
0%
Low CNOT7
High CNOT7
Pre
Post

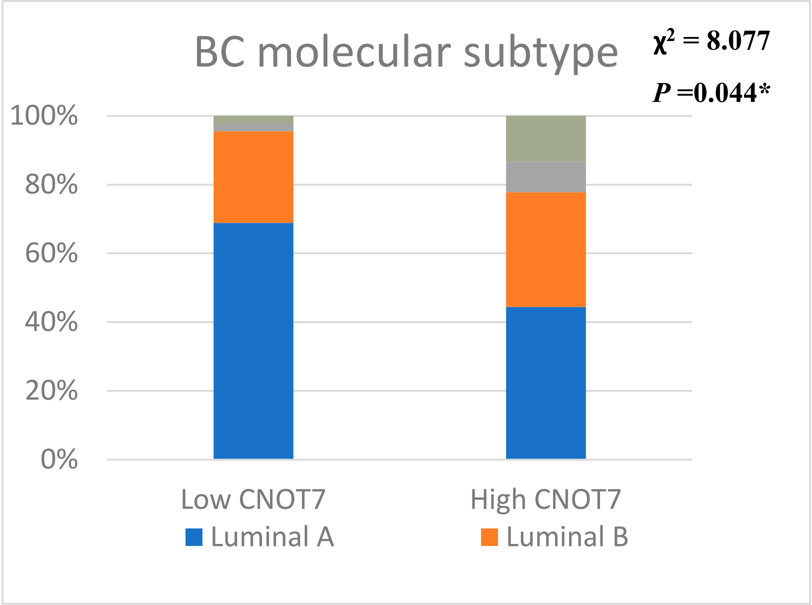
BC molecular subtype
χ2 = 8.077
P =0.044*
100%
80%
60%
40%
20%
0%
Low CNOT7
High CNOT7
Luminal A
Luminal B

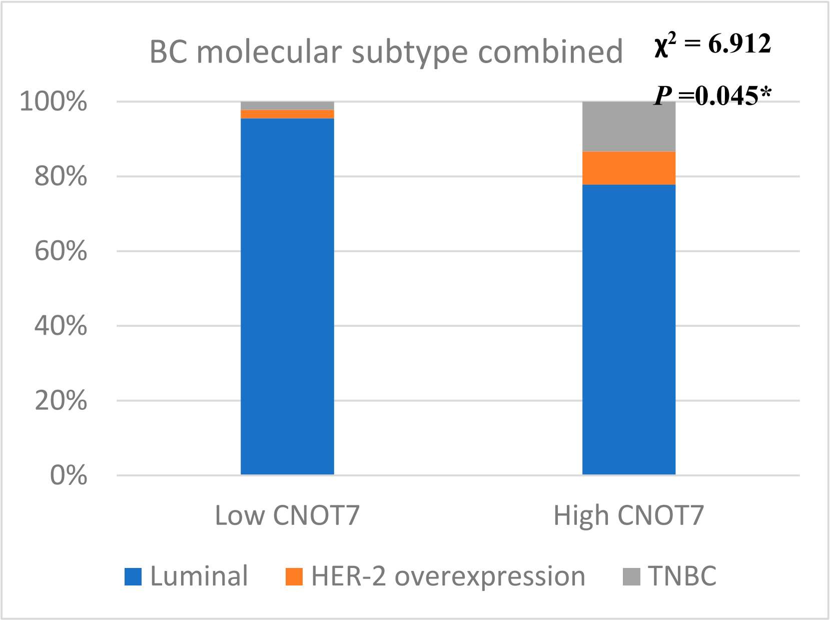
BC molecular subtype combined
χ2 = 6.912
P =0.045*
100%
80%
60%
40%
20%
0%
Low CNOT7
High CNOT7
Luminal
HER-2 overexpression
TNBC

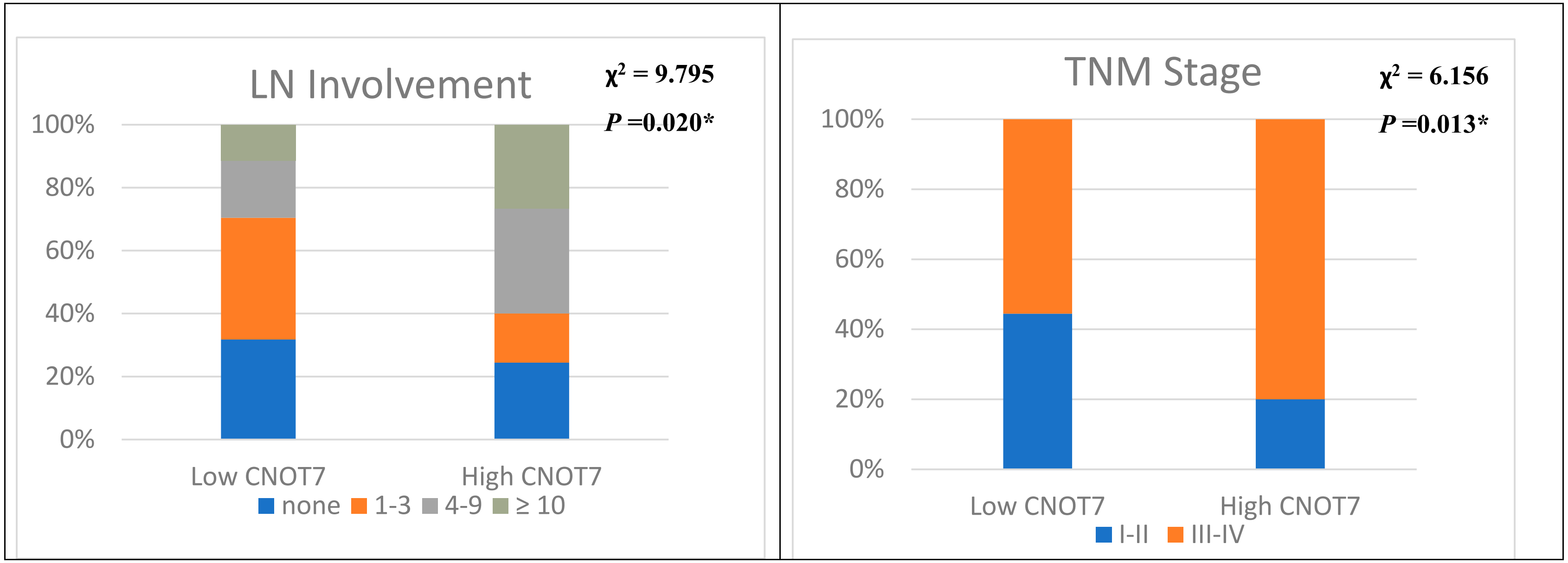
LN Involvement
χ2 = 9.795
P =0.020*
100%
80%
60%
40%
20%
0%
Low CNOT7
High CNOT7
none
1-3
4-9
≥ 10
TNM Stage
χ2 = 6.156
P =0.013*
100%
80%
60%
40%
20%
0%
Low CNOT7
High CNOT7
I-II
III-IV

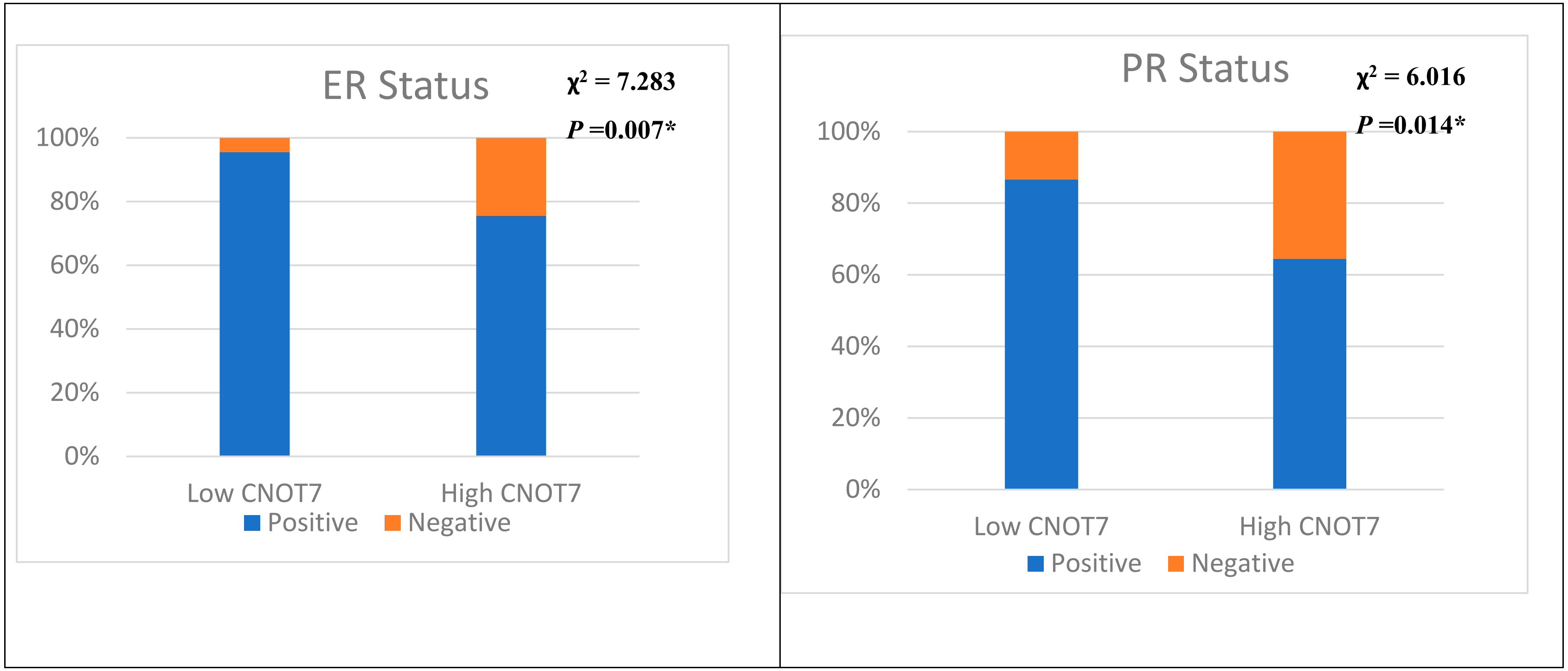
ER Status
χ2 = 7.283
P =0.007*
100%
80%
60%
40%
20%
0%
Low CNOT7
High CNOT7
Positive
Negative
PR Status
χ2 = 6.016
P =0.014*
100%
80%
60%
40%
20%
0%
Low CNOT7
High CNOT7
Positive
Negative

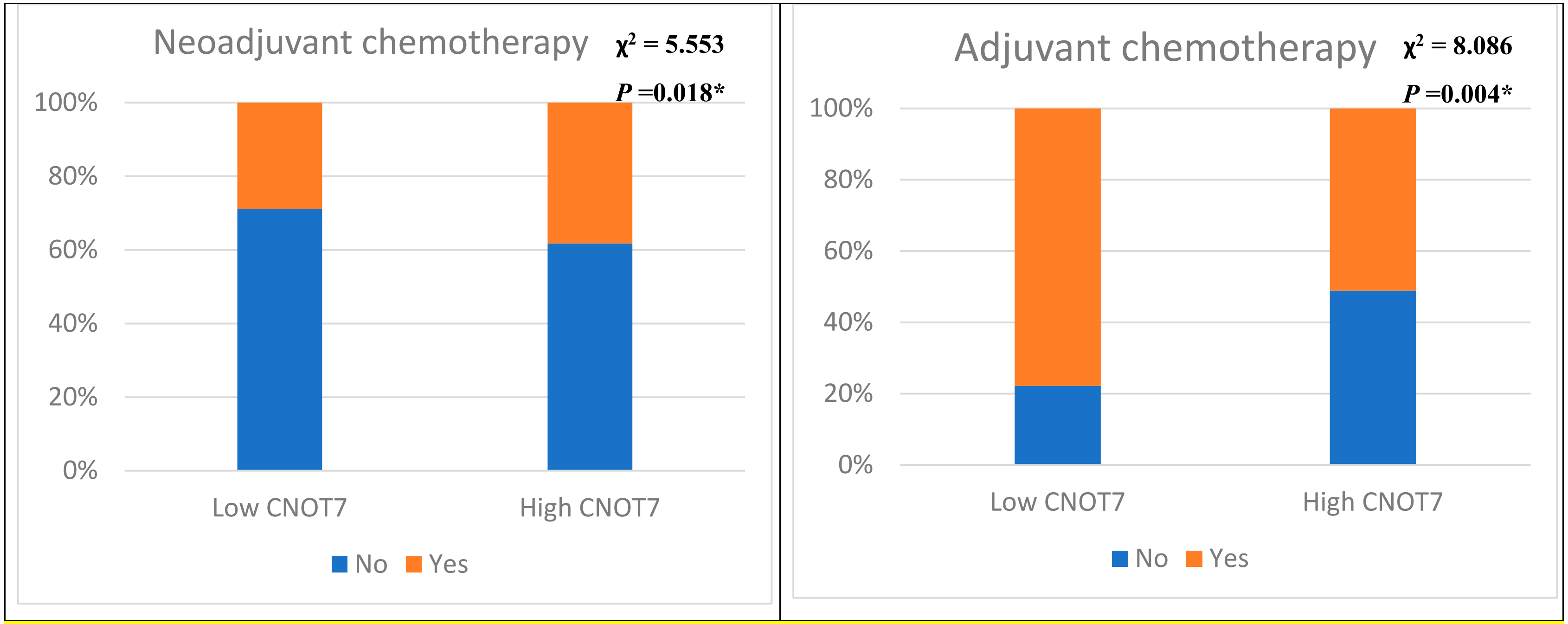

Figure. S4. Significant associations between CNOT7 serum levels (low and high) and clinicopathological variables in BC patients (n=90). Data are presented as count (n) and percentage (%). Statistical significance was determined using the Chi-square test (for dichotomous parameters), with significance set at $P<0.05$.

Significant associations were observed for BMI (P=0.048), Menopausal status (P=0.0005), BC molecular subtype (P=0.044), BC molecular subtype combined (P=0.045), ER status (P=0.007), PR status (P=0.014), LN involvement (P=0.020), TNM stage (P=0.013), neoadjuvant therapy (P=0.018) and adjuvant therapy (P=0.004).

Non-significant (NS) associations are not included in this figure, focusing only on the relationships identified as statistically significant in Table 2. [BMI, body mass index; BC, breast cancer; LN, lymph node; IDC, invasive ductal carcinoma; HER-2, human epidermal growth factor receptor 2; TNBC, triple negative breast cancer; ER, estrogen receptor; PR, progesterone receptor; TNM, tumor node metastasis.]

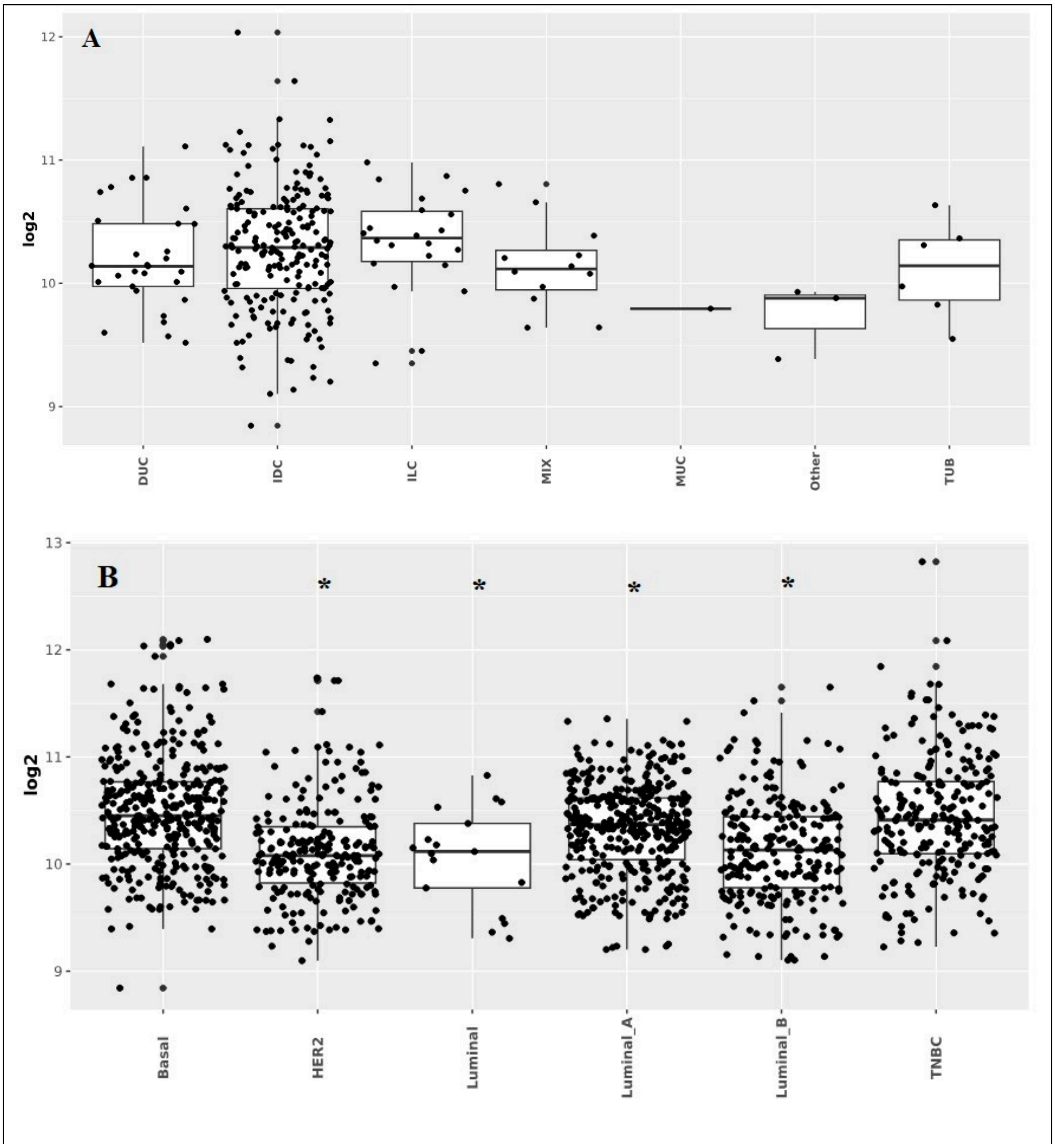
A
log2
12
11
10
9
DUC
IDC
ILC
MIX
MUC
Other
TUB
B
log2
13
12
11
10
9
*
*
*
*
Basal
HER2
Luminal
Luminal_A
Luminal_B
TNBC

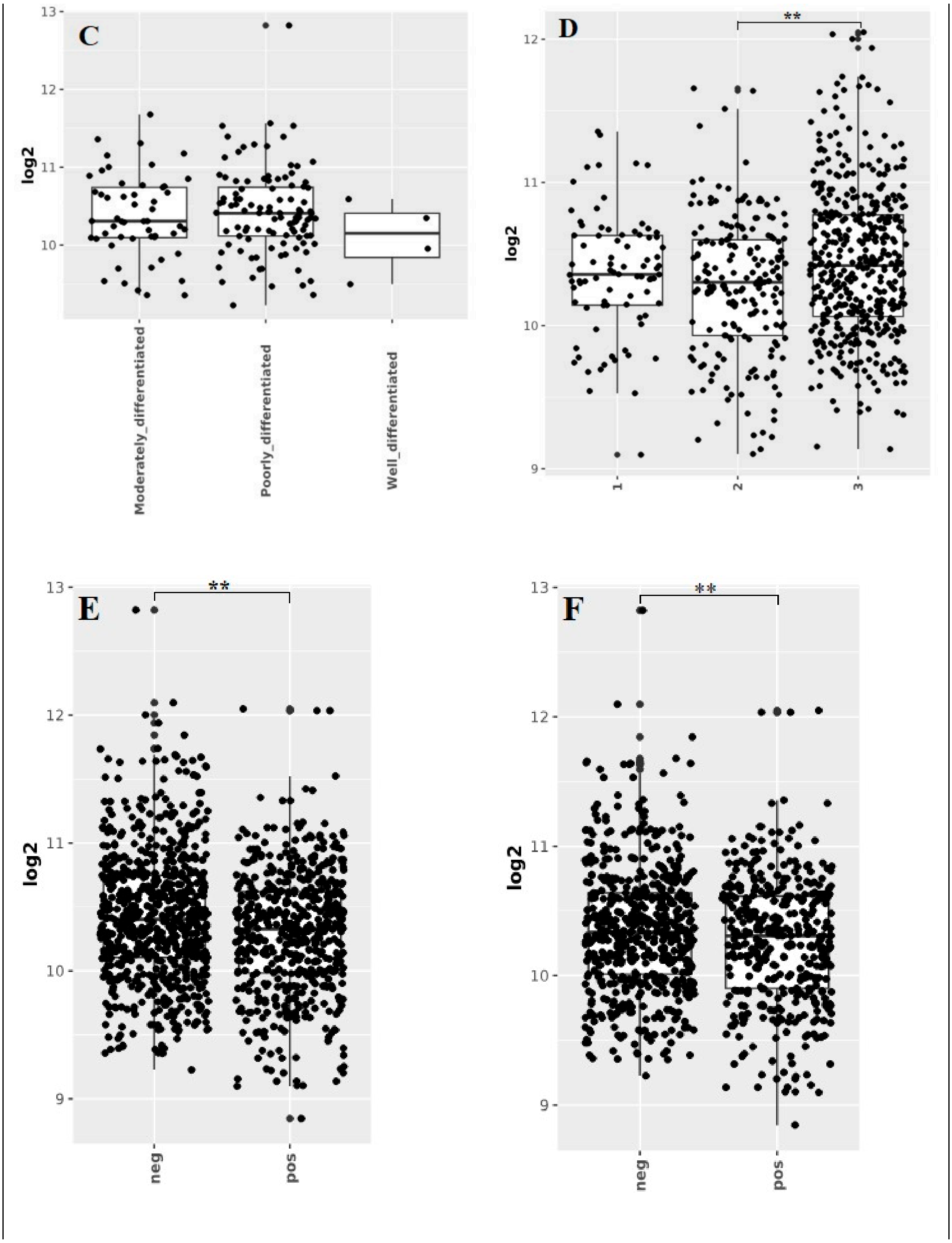
C
log2
13
12
11
10
Moderately_differentiated
Poorly_differentiated
Well_differentiated
D
**
log2
12
11
10
9
1
2
3
E
**
log2
13
12
11
10
9
neg
pos
F
**
log2
13
12
11
10
9
neg
pos

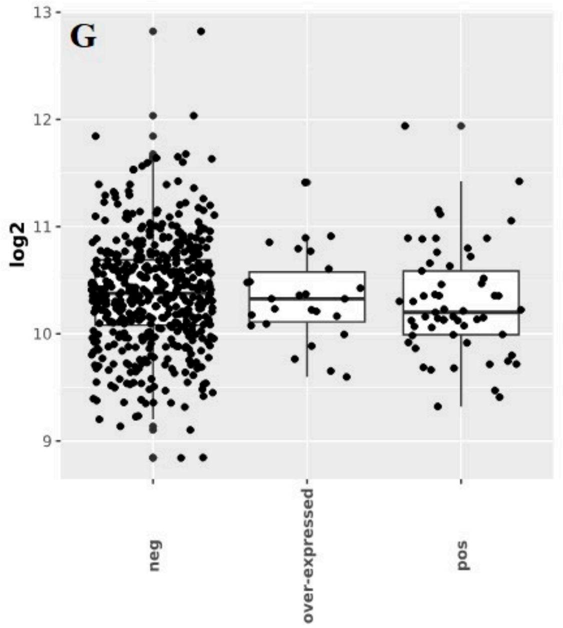

Figure. S5. CNOT7 expression profile in BC (A) histological subtypes **(B)** molecular subtypes **(C)** breast tissue histology **(D)** BC grade **(E)** estrogen receptor expression profile **(F)** progesterone receptor expression profile **(G)** human epidermal growth factor receptor 2 expression profile retrieved from http://gent2.appex.kr/gent2/ Accessed Feb. 8th, 2024. * Statistical significance is set at *P*< 0.05, ** Statistical significance is set at *P*< 0.01. [IDC, invasive ductal carcinoma; ILC, invasive lobular carcinoma; MUC, mucinous carcinoma; TUB, tubular carcinoma; HER2, human epidermal growth factor receptor 2; TNBC, triple negative breast cancer].

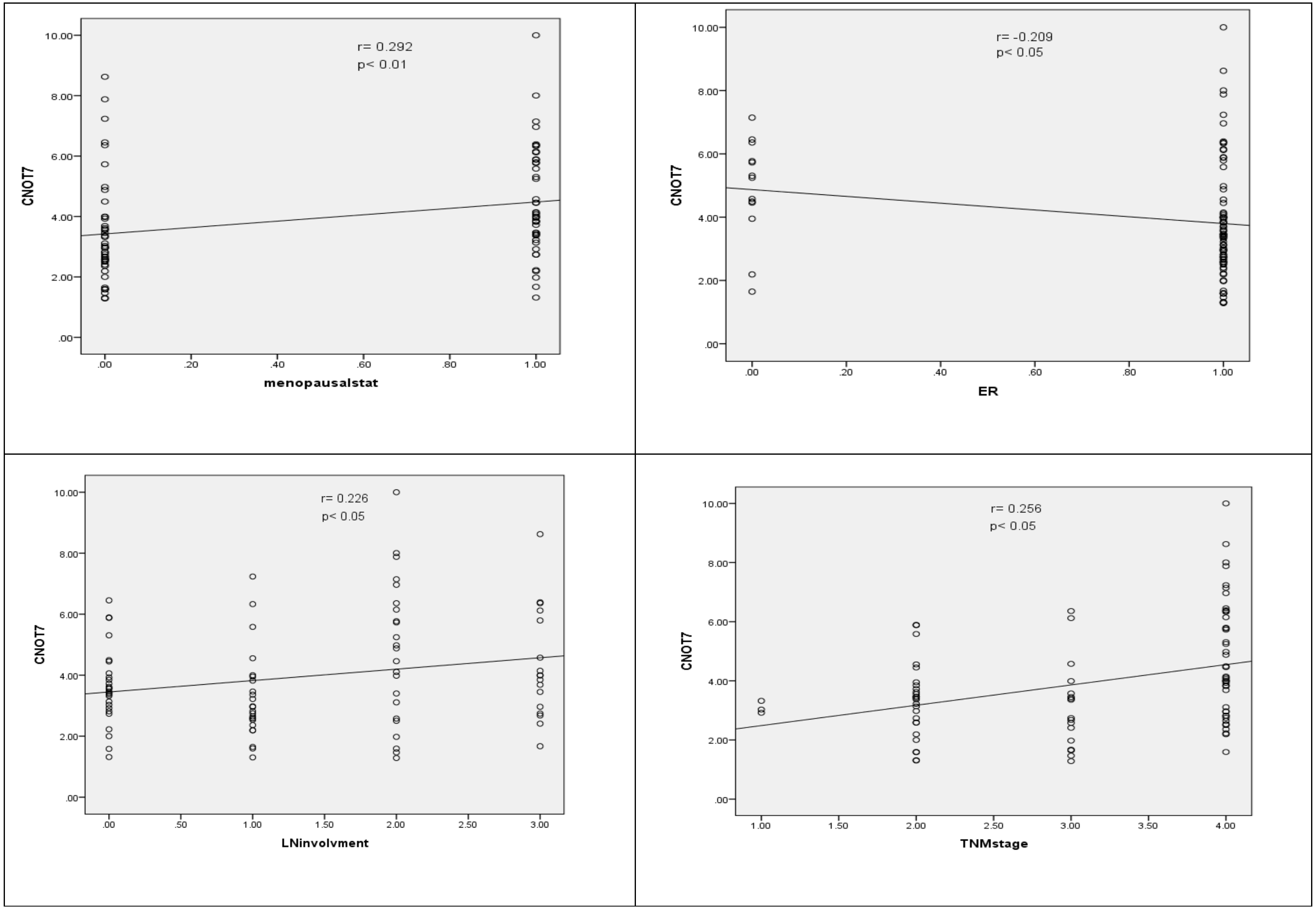

Figure. S6. CNOT7 serum levels correlations with BC patients clinicopathological characteristics where significant correlations were found for menopausal state, negative ER, LN involvement, and TNM late stages [ER; estrogen receptor;

TNM; tumor-node-metastasis.] Point-biserial correlation was used to measure the association that exists between two variables; one continuous and the other is dichotomous (used for the rest). *Significant statistical difference; *p*-value less than 0.05.
